# Dynamic relationships between body size, species richness, abundance, and energy use in a shallow marine epibenthic faunal community

**DOI:** 10.1002/ece3.1343

**Published:** 2014-12-28

**Authors:** Fabio A Labra, Eduardo Hernández-Miranda, Renato A Quiñones

**Affiliations:** 1 Centro de Investigación e Innovación para el Cambio Climático, Facultad de Ciencias, Universidad Santo TomasEjercito 146, Código Postal, 8370003, Santiago, Chile; 2 Programa de Investigación Marina de Excelencia (PIMEX), Facultad de Ciencias Naturales & Oceanográficas, Universidad de ConcepciónConcepción, Chile; 3 Interdisciplinary Center for Aquaculture Research (INCAR), Casilla 160-C, Universidad de ConcepciónConcepción, Chile

**Keywords:** Coliumo bay, energetic equivalence, humboldt current system, hypoxia, macroecological dynamics, south eastern pacific

## Abstract

We study the temporal variation in the empirical relationships among body size (S), species richness (R), and abundance (A) in a shallow marine epibenthic faunal community in Coliumo Bay, Chile. We also extend previous analyses by calculating individual energy use (E) and test whether its bivariate and trivariate relationships with S and R are in agreement with expectations derived from the energetic equivalence rule. Carnivorous and scavenger species representing over 95% of sample abundance and biomass were studied. For each individual, body size (g) was measured and E was estimated following published allometric relationships. Data for each sample were tabulated into exponential body size bins, comparing species-averaged values with individual-based estimates which allow species to potentially occupy multiple size classes. For individual-based data, both the number of individuals and species across body size classes are fit by a Weibull function rather than by a power law scaling. Species richness is also a power law of the number of individuals. Energy use shows a piecewise scaling relationship with body size, with energetic equivalence holding true only for size classes above the modal abundance class. Species-based data showed either weak linear or no significant patterns, likely due to the decrease in the number of data points across body size classes. Hence, for individual-based size spectra, the SRA relationship seems to be general despite seasonal forcing and strong disturbances in Coliumo Bay. The unimodal abundance distribution results in a piecewise energy scaling relationship, with small individuals showing a positive scaling and large individuals showing energetic equivalence. Hence, strict energetic equivalence should not be expected for unimodal abundance distributions. On the other hand, while species-based data do not show unimodal SRA relationships, energy use across body size classes did not show significant trends, supporting energetic equivalence.

## Introduction

Understanding how body size influences observed patterns in species abundance and diversity has been a long-standing area of research in ecology (Peters 1983; Calder 1984; Brown et al. 2004; White et al. 2007; Sibly et al. 2012). A central focus in these efforts has been the study of the bivariate relationship between abundance (often measured as density) and body size across species, and its implications for the way energy and resources are partitioned in communities or species assemblages (Damuth 1981; Maurer and Brown 1988; Marquet et al. 1990; Enquist et al. 1998; Allen et al. 2002; Brown et al. 2004; White et al. 2007; Sibly et al. 2012). Analyses of global compilations of data on the average body size of mammal species and their average population density have shown a power law scaling relationship (Damuth 1981, 1987). This global size–density relationship (GSDR) showed a scaling exponent that is the inverse of the exponent of the relationship between body size and metabolic rate (Damuth 1981, 1987; White et al. 2007), which implies that the energy flux per unit area in a given population, estimated as the product of metabolic rate and abundance, will be independent of body mass. Hence, this particular scaling of abundance was referred to as the energetic equivalence rule (EER) by Nee et al. (1991). Different authors have tried to establish the generality of the predicted energetic equivalence across species of different sizes, with species-based data from local communities presenting much broader scatter in the bivariate relationship, which led to the suggestion that local size–density relationships (LSDR) may be better described by polygonal or quantile regressions (Damuth 1981; Maurer and Brown 1988; Marquet et al. 1995; White et al. 2007). The variability observed in global or local species-based scaling relationships contrasts with the results obtained when individual-based analyses are carried out (Enquist et al. 1998). In particular, examination of individual size distributions (ISD), or the frequency distribution of individual plant sizes across all organisms in a community, regardless of their species, has provided strong empirical support for the energetic equivalence (Enquist et al. 1998; Enquist and Niklas 2001). This individual-based approach allows different species to take more than one body size value, and as a result, the abundance is estimated across the community or ensemble as a whole rather than for each separate species (White et al. 2007). Thus, individual-based scaling patterns have provided renewed support for the EER (Enquist et al. 1998; Enquist and Niklas 2001), becoming one of the basic assumptions of the metabolic theory of ecology (MTE, Allen et al. 2002; Brown et al. 2004; Sibly et al. 2012). Nevertheless, recent work has questioned the generality of EER, suggesting it should be considered a null hypothesis rather than a rule (Isaac et al. 2011, 2013). These authors suggested that greater emphasis should be given to understanding the mechanisms underlying abundance–energy relationships and to consider alternative (i.e., nonenergetic) determinants of species abundance (Isaac et al. 2013).

To date, most work on the scaling of species abundance has not considered the interaction of species body size and abundance with other community level variables such as species richness. Early studies by May (1988) and Morse et al. (1985) proposed that these three variables, species richness, abundance, and body size, are interrelated, suggesting that any efforts to provide a unifying theory for these community attributes should consider all three of them. Siemann et al. (1996, 1999) showed for grassland insects that the relationship between body size (measured as biovolume), species richness, and abundance (measured as the number of individuals) follows a parabola in these three dimensions, with the number of species across size classes scaling as a power law of the number of individuals in these size classes. Studies examining the univariate frequency distributions of the number of species across body size classes have shown that this typically follows a unimodal, right-skewed shape even after logarithmic transformation (Gaston and Blackburn 2000; Blackburn and Gaston 2002). The SRA pattern extends these analyses by taking into account the number of individuals present in different body size classes (Siemann et al. 1996, 1999). In their studies on grassland insects, Siemann et al. (1996, 1999) examined the frequency distribution of individuals (or species) across different body size classes, using data on the oldest life stage for each of the species observed in sweep net samples, so that each species may occupy a single body size class. A similar species-based approach was taken by Fa and Fa (2002), who calculated each species' average body size using all sampled individuals in multitaxon assemblage of marine mollusks. Later work by McClain (2004) examined the generality of the SRA pattern in deep-sea gastropods by examining individual-based data, thus allowing each species to potentially occupy more than one body size class (McClain 2004). The decision to focus on species-based or individual-based analyses allows different research questions to be addressed. The use of species-based analyses allows researchers to examine questions on the evolution of species body size and whether the processes that constrain it may operate in taxa with different evolutionary histories (Siemann et al. 1996, 1999; Fa and Fa 2002). On the other hand, examination of individual-based frequency distributions allows focus to be placed on the ecological and demographic processes taking place within the community, with body size being a dynamic characteristic that may change during the ontogeny, rather than a morphological trait subject to evolutionary change.

Individual size distributions have also been referred to as size spectra (Kerr and Dickie 2001; White et al. 2007). In addition to unimodal (Siemann et al. 1996; McClain 2004) and power law size spectra (Enquist et al. 1998; Kerr and Dickie 2001), multimodal size spectra may be observed, where the distribution of individuals of different sizes is characterized by multiple peaks (e.g., Sheldon et al. 1972; Griffiths 1986; Havlicek and Carpenter 2001). Studies documenting the power law or multimodal size spectra usually focus on the bivariate abundance–body size pattern, examining its implications for energy use and partition, but neglect the interaction with species richness. On the other hand, studies documenting the trivariate SRA relationship do not examine the interaction of species richness, abundance, and body size from an energetic viewpoint. One possible reason is that studies of the SRA relationship have often relied either on body length (e.g., Morse et al. 1985; May 1988) or on biovolume, which is calculated based on an organism's linear dimensions through a combination of length, width, and height (Siemann et al. 1996, 1999; Fa and Fa 2002; McClain 2004). In addition, studies documenting the trivariate SRA relationship have often focused on assemblages of a given taxonomic group, such as terrestrial arthropods (Morse et al. 1985; Siemann et al. 1996, 1999) and marine mollusks (Fa and Fa 2002; McClain 2004). In all these systems, observed SRA relationships tend to follow a parabola, with logarithmic transformations of both abundance and species richness showing a right-skewed unimodal pattern with body size, and species richness scaling with the number of individuals (Siemann et al. 1996, 1999; Fa and Fa 2002; McClain 2004). In order to fit SRA relationships, individuals are classified into base-2 logarithmic body size bin intervals (Siemann et al. 1996, 1999; Fa and Fa 2002; McClain 2004), a procedure that has yielded unimodal size distributions. This pattern differs from the EER expectation, where the number of individuals for different body size classes should follow a power law relationship, showing a linear scaling on double-log axes (Enquist et al. 1998; Enquist and Niklas 2001; White et al. 2007, 2008). Also, while biovolume is a simple measure to calculate, it does not necessarily correlate well with an organism's body mass and hence does not readily allow the estimation of organism energy use through allometric scaling.

In this contribution, we examine the trivariate SRA relationship in a shallow epibenthic marine community from an energetic perspective, comparing the patterns obtained using both individual-based and species-based descriptions the SRA relationship. Specifically, we aim to examine the generality of the SRA relationship and its interaction with patterns of energy use. Hence, in order to understand the importance of energy flow in structuring this community, we also examine the scaling of total energy use (E) across body size bins and examine the relationship between body size and species richness with energy (SRE). To do so, we focus on body mass as our estimate of body size rather than on particular linear dimensions or biovolume. This allows us to examine whether individuals belonging to different size classes differ or not in their overall energy use. Under the EER (Damuth 1981; Nee et al. 1991; Marquet et al. 1995), the null expectation is that total energy use would be invariant with body size. Following traditional tests of this prediction, abundance should scale negatively with body size (Damuth 1981; Nee et al. 1991; Enquist et al. 1998; White et al. 2007, 2008), rather than exhibit a unimodal right-skewed relationship (Siemann et al. 1996, 1999). In addition, if energetic equivalence holds true, total energy use in each size bin should be equal or very similar across all size bins. Finally, to ascertain the generality of the observed patterns, we also examine their temporal variability, using ecological monitoring data collected at 3-month intervals during 2 years. This data set allows us to assess the temporal changes in both SRA and SRE relationships, using both species and individual-based estimates of body size.

## Methods

### Study area and sampling

Coliumo Bay (36°32′S; 72°56′W) is a small bay with depths shallower than 25 m, characterized mainly by sandy or sedimentary substrate. Data were collected as part of the research program set up by the Programa de Investigación Marina de Excelencia (PIMEX) at the Universidad de Concepción (UDEC), Chile. Periodic sampling of epibenthic (macro and mega) fauna was performed at 3-month intervals. Samples were obtained during the months of January, May, August, and November 2007, and January, April, July, and October 2008, using UDEC's research vessel L/C Kay–Kay II, at three sampling stations within Coliumo Bay using a modified Agassiz trawl (1 m wide × 1 m long × 30 cm high, lined with 5 mm “knot” to “knot” netting). Survey distance averaged 378 ± 145 m, cruising for 5 min at 1.5–2 knots. Once collected, biological samples were washed and separated on a 500-*μ*m mesh. Trawl sampling techniques have been used to describe and quantify epibenthic macro- and megafaunal communities also demersal fish species in several environments (e.g., McKnight and Probert 1997; Callaway et al. 2007; Griffiths et al. 2008; : Hernández-Miranda et al. 2012a,b).

### Analyses

All individuals were identified to the species level using taxonomic keys available for the study area. In those cases in which species could not be determined, individuals were identified to the genus level. Species were assigned to different feeding guilds following Gutiérrez et al. (2000) and Laudien et al. (2007). Here, we focused on carnivorous and scavenger species, which accounted for over 95% of the observed individuals. So in this study, macro- and megafaunal species correspond to carnivorous and scavenger organisms with body size ranging from 0.5 mm to ca 300 mm (See Table S1, Appendix S1 Supporting Information for a detail of the included taxa). This allowed us to focus on species belonging to well-represented functional groups of macro- and megafaunal community. Each individual was measured and weighed using a precision analytic balance (wet weight in grams, 0.001 g sensitivity). Individual energy use was estimated for every individual, using the measured body weight and allometric equations available in the literature for each taxonomic group (Peters 1983; Alekseeva and Zotin 2001; Vladimirova 2001; Vladimirova et al. 2003). The relationships between abundance (A), species richness (S), energy use (E), and body size were assessed in a similar manner to previous studies (Siemann et al. 1996, 1999). Briefly, we summed and log-transformed the number of species, the number of individuals, and total energy use across different log_e_ weight classes. Weight classes were defined using the progression class(*i*) = exp(*i*), with *i* = −9, −8,…,8; obtaining equidistant body size classes on a logarithmic scale which encompass the observed range of individual weights. Once these weight classes were defined, the total number of individuals, total number of species, and the total amount of energy use within each class were calculated. While other studies have used alternative approaches such as kernel smoothing methods (McClain 2004), we aimed to allow easier comparison of our results and hence followed the approach described by Siemann et al. (1996, 1999). We examined univariate relationships between species richness and abundance and log size classes by fitting a four-parameter Weibull function. The specific form used was:

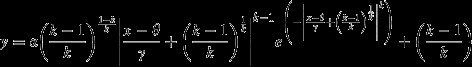
1

where *α* > *0* is a normalization parameter, *γ *> *0* is the scale parameter, *k > 0* is the shape parameter and *θ* is the location parameter (Johnson et al. 1994; Evans et al. 2000; SigmaPlot 2004. SigmaPlot for Windows. Ver. 9. Systat Software, Point Richmond, CA). Weibull function fits were carried out using nonlinear least-squares regression in the R program (R Development Core Team 2008, available at www.r-project.org). Given that most macroecological studies have focused on the existence of negative power law scaling relationships between abundance and body size, we also tested the alternative hypothesis that abundance scales with body size following a power law:

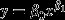
2

where *β*_0_ and *β*_1_ are the power law normalization constant and exponent parameters, respectively (White et al. 2007, 2008). It is important to note that EER as a null hypothesis assumes that the power law exponent takes the reciprocal value to the allometric power law exponent relating metabolic rate with body size (Damuth 1981, 1987; Nee et al. 1991). We compared the performance of Weibull and power law relationships using the Bayesian information criterion (BIC or Schwarz Criterion; Schwarz 1978), selecting the minimum BIC to determine the best model.

To characterize the number of species as a function of the number of individuals in each size class, we followed previous studies and fitted a power function so that

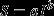
3

where *a* and *b* are the power law parameters (Siemann et al. 1996, 1999; Fa and Fa 2002; McClain 2004), which were fitted using OLS regression on log-transformed data. To characterize the dependence of log energy use within each size class as a function of both size and the number of individuals in each size class, we tested whether linear or piecewise linear regressions best fit the scaling of log(energy use) as a function of log(body size). Following Muggeo (2003), a piecewise or segmented relationship between the mean response *μ *= *E*[*Y*] and the variable *X*, for observation *i* = 1, 2,…, *n*, was modeled by adding the following terms in the linear predictor:

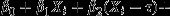
4

where (*X_i_* − *τ*) + = (*X_i_* − *τ*) × *I*(*X_i_* > *ψ*) and *I*(·) is an indicator function that is equal to one when the statement is true and is equal to zero when the statement is false (Muggeo 2003). Piecewise linear models were fitted using the segmented library in the R program (R Development Core Team 2008, available at www.r-project.org) and ranked according to the Bayesian information criterion (BIC or Schwarz Criterion; Schwarz 1978). Again, minimum BIC was selected to determine the best model between linear and piecewise linear relationships.

## Results

Monitoring of the epibenthic macro–megafaunal community of Coliumo Bay from January 2007 to October 2008 with trawl sampling gear allowed us to capture, identify, measure, and weigh 36726 individuals. The data show this to be a diverse community, with representation of four phyla and five classes (Asteroidea, Elasmobranchii, Actinopterygii, Malacostraca, and Gastropoda). Over the whole study period, we observed a total of 39 species belonging to 13 orders and 32 families (see Appendix S1 in Supporting Information). Despite the observed taxonomic diversity, individual-based data from the epibenthic fauna at Coliumo Bay showed unimodal right-skewed relationships between abundance and the body size classes, as well as between species richness and body size. This may be appreciated when we examine data for January 2007 (Fig.A and B; Table1). Total energy use and abundance across individual-based size classes showed a *piecewise* scaling relationship, with energy use increasing with body size up to the modal size class (Fig.C; Table2), while the relationship between species richness and abundance showed a power function with a scaling exponent of 0.33 (Fig.D; Table3). In this sample, the relationship between abundance and energy use showed a significant but weak pattern (OLS linear regression *A* = −0.814 + 1.21E, *R*^2^ = 0.39, *P* = 0.0068). However, scaling relationships were observed for body size classes below and above the modal size class (Fig.E). A similar pattern is observed for species richness and energy use, with data points below and above the modal size class showing different trends. Nevertheless, in this case, the overall pattern may be well fit by a polynomial function (*S* = 0.88 + 0.168E − 0.007E^2^) (Fig.F). When species-averaged data are examined, the unimodal abundance and richness patterns are no longer observed, and linear scaling relationships describe the bivariate relationships examined (Fig.2; Tables6).

**Table 1:** Relationship between (A) log abundance and individual log body size, (B) log species richness and individual log body size. The table shows the estimated values for the 4-parameter Weibull function and the linear (power law) fit for body size. Also shown are the Bayesian Information Criterion (BIC) and *R*^2^ values for each model. Minimum values of BIC are highlighted in bold, indicating the selected model

Model	Linear				Weibull					
Date	*β*_0_	*β*_1_	BIC	*R* ^2^	*θ*	*α*	*γ*	*κ*	BIC	*R* ^2^
(A)										
January 2007	3.14±0.47^*^^*^^*^	−0.04±0.11^ns^	61.05	0.01	−0.43±0.35^ns^	5.27±0.25^*^^*^^*^	7.89±1.17^*^^*^^*^	2.27±0.43^*^^*^^*^	**47.52**	0.92
May 2007	3.37±0.55^*^^*^^*^	0.1±0.13^ns^	71.28	0.04	−0.38±0.19^ns^	6.1±0.18^*^^*^^*^	7.2±0.26^*^^*^^*^	1.94±0.12^*^^*^^*^	**51.94**	0.97
August 2007	3.66±0.52^*^^*^^*^	−0.11±0.13^ns^	57.77	0.06	−2.12±1.1^ns^	5.46±0.57^*^^*^^*^	7.15±1.29^*^^*^^*^	1.28±0.32^*^^*^^*^	**44.04**	0.59
November 2007	5.04±0.33^*^^*^^*^	−0.16±0.08^ns^	45.74	0.18	−1.66±0.78^ns^	6.17±0.33^*^^*^^*^	9.67±1.23^*^^*^^*^	1.38±0.22^*^^*^^*^	**36.15**	0.76
January 2008	3.87±0.6^*^^*^^*^	−0.03±0.12^ns^	84.52	0.01	−1.51±0.35^*^^*^^*^	7.09±0.37^*^^*^^*^	10.6±1.91^*^^*^^*^	2.92±0.61^*^^*^^*^	**64.78**	0.9
April 2008	0.6±6.31^*^^*^^*^	0.13±0.01^ns^	72.87	0.01	−1.55±0.86^ns^	6.22±0.49^*^^*^^*^	7.6±1.14^*^^*^^*^	1.32±0.19^*^^*^^*^	**51.35**	0.82
July 2008	4.77±0.68^*^^*^^*^	−0.23±0.16^ns^	69.87	0.08	−3.52±1.6^ns^	8.22±2.17^*^^*^^*^	7.97±2.59^*^^*^	1.05±0.19^*^^*^^*^	**30.97**	0.67
October 2008	4.36±0.69^*^^*^^*^	−0.14±0.16^ns^	70.34	0.06	−2.8±1.63^ns^	8.42±3.87^ns^	7.26±2.66^*^^*^	1.03±0.21^*^^*^^*^	**51.75**	0.67
(B)										
January 2007	1.22±0.16^*^^*^^*^	0.05±0.04^ns^	35.02	0.07	0.11±0.27^ns^	1.94±0.08^*^^*^^*^	9.63±0.69^*^^*^^*^	2.25±0.24^*^^*^^*^	−**3.05**	0.95
May 2007	1.11±0.22^*^^*^^*^	0.03±0.05^ns^	43.36	0.02	−0.76±0.42^ns^	2.09±0.16^*^^*^^*^	6.81±0.49^*^^*^^*^	1.77±0.17^*^^*^^*^	**16.72**	0.89
August 2007	1.22±0.17^*^^*^^*^	−0.01±0.04^ns^	28.86	0.08	−2.15±2.11^ns^	1.67±0.23^*^^*^^*^	9.66±4.52^ns^	1.21±0.48^*^	**21.26**	0.63
November 2007	1.64±0.15^*^^*^^*^	−0.02±0.04^ns^	26.05	0.02	0.00±0.64^ns^	2.15±0.13^*^^*^^*^	9.42±1.32^*^^*^^*^	1.91±0.49^*^^*^	**11.56**	0.78
January 2008	1.17±0.15^*^^*^^*^	−0.06±0.03^ns^	31.76	0.12	0.38±0.59^ns^	1.78±0.15^*^^*^^*^	14.25±6.8^ns^	3.85±1.97^ns^	**16.41**	0.79
April 2008	1.4±0.21^*^^*^^*^	−0.05±0.05^ns^	34.41	0.01	−1.09±0.35^*^^*^	2.17±0.13^*^^*^^*^	6.75±0.42^*^^*^^*^	1.67±0.12^*^^*^^*^	**21.12**	0.87
July 2008	1.24±0.23^*^^*^^*^	0.06±0.05^ns^	39.03	0.02	1.23±0.71^ns^	2.04±0.22^*^^*^^*^	8.51±1.21^*^^*^^*^	1.75±0.26^*^^*^^*^	**27.07**	0.73
October 2008	1.33±0.22^*^^*^^*^	−0.01±0.05^ns^	32.83	0.01	0.02±0.37^ns^	2.07±0.09^*^^*^^*^	6.74±0.41^*^^*^^*^	1.49±0.13^*^^*^^*^	**1.34**	0.94

Significance results for the slope parameters are shown by the following abbreviations: ^*^^*^^*^P < 0.001; ^*^^*^P < 0.01; ^*^P<0.05; ns: *P* > 0.05. See text for details on the meaning of the different parameters.

**Table 2:** Relationship between log energy use and individual log body size. We tested the fit of the linear and piecewise scaling models for all taxa. The table shows the fitted values of the intercept (*β*_0_), slope of the first scaling region (*β*_1_), slope of the second scaling region (*β*_2_) and the threshold value (*τ*). Also shown are the Bayesian Information Criterion (BIC) and *R*^2^. Minimum values of BIC are highlighted in bold, indicating the selected model

Model	Linear				Piecewise					
Date	*β*_0_	*β*_1_	BIC	*R* ^2^	*β*_0_	*β*_1_	*β*_2_	*τ*	BIC	*R* ^2^
All taxa										
January 2007	2.83±0.49^*^^*^^*^	0.57±0.11^*^^*^^*^	67.79	0.67	5.86±0.43^*^^*^^*^	1.25±0.08^*^^*^^*^	−0.14±0.08^ns^	−0.38±0.41	**34.75**	0.97
May 2007	3.44±0.50^*^^*^^*^	0.59±0.12^*^^*^^*^	68.66	0.64	6.50±0.44^*^^*^^*^	1.40±0.10^*^^*^^*^	−0.22± 0.08^ns^	−0.03±0.34	**31.8**	0.97
August 2007	3.66±0.42^*^^*^^*^	0.42±0.10^*^^*^	57.75	0.57	8.02±1.68^*^^*^^*^	1.46±0.34^*^^*^	0.11±0.11^ns^	−2.36±0.83	**52.06**	0.81
November 2007	4.96±0.30^*^^*^^*^	0.37±0.07^*^^*^^*^	44.29	0.63	9.74±2.07^*^^*^	1.72±0.50^*^^*^	0.18±0.08^*^	−0.26±0.59	**36.3**	0.83
January 2008	4.57±0.63^*^^*^^*^	0.18±0.16^ns^	63.17	0.02	16.78±1.97^*^^*^^*^	3.63±0.48^*^^*^^*^	−0.27±0.07^*^	−2.72±0.22	**35.03**	0.91
April 2008	4.83±0.38^*^^*^^*^	0.37±0.11^*^^*^	43.76	0.54	12.36±3.64^*^^*^	2.88±1.04^*^	0.17±0.08^ns^	−2.51±0.46	**34.19**	0.87
July 2008	5.64±0.40^*^^*^^*^	0.15±0.11^ns^	**44.8**	0.09	14.29±1.04^ns^	2.82±1.04^ns^	0.05±0.08^ns^	−3.00±0.47	75.09	0.18
October 2008	5.08±0.57^*^^*^^*^	0.24±0.15^ns^	53.76	0.12	19.88±5.76^*^^*^	4.93±1.63^*^	−0.02±0.13^ns^	−2.80±0.32	**45.01**	0.65

Significance levels for the slope parameters are shown by the same abbreviations as in Table1.

**Table 3:** Relationship between log species richness and log abundance within individual based body size classes. We tested the fit of the linear model for all taxa. The table shows the fitted values of the intercept (*β*_0_) and slope of the scaling region (*β*_1_). For the slope parameter, the 95% confidence intervals are shown in parentheses rather than standard errors (see text for details on theoretical expectations). Also shown are the Bayesian Information Criterion (BIC) and *R*^2^

Model	Linear			
Date	*β*_0_	*β*_1_	BIC	*R* ^2^
All taxa				
January 2007	0.2382^ns^	0.3347 (0.26–0.59)^*^^*^^*^	9.38	0.83
May 2007	−0.1241^ns^	0.3668 (0.29–0.66)^*^^*^^*^	11.42	0.87
August 2007	0.2549^ns^	0.2696 (0.17–0.44)^*^^*^^*^	12.77	0.68
November 2007	0.1626^ns^	0.2985 (0.13–0.43)^*^^*^	16.46	0.49
January 2008	0.1969^ns^	0.197 (0.1–0.29)^*^^*^	33.97	0.45
April 2008	−0.1229^ns^	0.3421 (0.25–0.59)^*^^*^	19.79	0.79
July 2008	0.7338^ns^	0.1352 (−0.03–0.22)^ns^	37.64	0.11
October 2008	0.3211^ns^	0.2138 (0.09–0.31)^*^^*^	27.96	0.46

Significance levels for the slope parameters are shown by the same abbreviations as in Table1.

**Table 4:** Relationship between (a) log abundance and log species-averaged body size data, (b) log species richness and log species-averaged body size data. The table shows the estimated values for the 4-parameter Weibull function and the linear (power law) fit. In those cases where parameter estimates did not converge, no parameter values are shown and are indicated with a dash symbol (–). Also shown are the Bayesian Information Criterion (BIC) and *R*^2^ values. Minimum values of BIC are highlighted in bold, indicating the selected model

Model	Linear				Weibull					
Date	*β*_0_	*β*_1_	BIC	*R* ^2^	*θ*	*α*	*γ*	*κ*	BIC	*R* ^2^
(A)										
January 2007	1.47±0.59^*^	−0.17±0.21^ns^	**24.65**	0.11	−2.5±6E08^ns^	5±7.75^ns^	2.4±6.66^ns^	1±1E09^ns^	31.66	0.04
May 2007	1.67±0.50^*^	−0.11±0.21^ns^	**26.28**	0.06	−2.5±2E08^ns^	5±2.73^ns^	3.26±1.62^ns^	1±4E08^ns^	26.58	0.50
August 2007	1.64±0.65^*^	−0.11±0.21^ns^	**23.86**	0.06	11.3±2E16^ns^	5±2E16^ns^	10±2E16^ns^	3.3±3E14^ns^	33.5	0.30
November 2007	1.83±0.56^ns^	−0.13±0.20^ns^	31.81	0.07	−2.5±1E08^ns^	5±3.34^ns^	4.1±0.99^ns^	1±1E08^ns^	**28.35**	0.64
January 2008	2.19±0.33^ns^	−0.25±0.15^ns^	**45.12**	0.20	−2.5±7E07^ns^	5±3.03^ns^	3.6±0.52^ns^	1±1E08^ns^	57.97	0.12
April 2008	2.72±0.49^ns^	−0.38±0.21^ns^	**15.07**	0.52	−2.5±2E08^ns^	5±10.64^ns^	4.5±2E05^ns^	1±1E08^ns^	19.13	0.48
July 2008	2.90±0.43^ns^	−0.43±0.15^ns^	**20.25**	0.61	−2.5±7E07^ns^	5±3.50^ns^	4.5±0.90^ns^	1±7E07^ns^	23.10	0.67
October 2008	2.75±0.59^ns^	−0.38±0.23^ns^	**21.17**	0.40	−2.5±2E08^ns^	5±4.67^ns^	4.12±1.37^ns^	1±1E08^ns^	23.59	0.52
(B)										
January 2007	0.32±0.18^ns^	−0.03±0.07^ns^	8.47	0.05	11.25^ns^	5^a,ns^	1^a,ns^	1^a,ns^	**–**	**–**
May 2007	0.35±0.12^*^	0.01±0.05^ns^	5.77	0.00	−2.5±9E09^ns^	5±34.96^ns^	1±12.34^ns^	1±49E10^ns^	**–**	**–**
August 2007	0.31±0.14^ns^	−0.01±0.05^ns^	5.62	0.01	11.25^ns^	7.5^ns^	9.053^a,ns^	2^a,ns^	**–**	**–**
November 2007	0.3±0.1^*^	0.02±0.04^ns^	4.52	0.03	11.25^ns^	5^a,ns^	1^a,ns^	1^a,ns^	**–**	**–**
January 2008	0.31±0.06^*^^*^^*^	0.01±0.03^ns^	0.92	0.01	−2.5±7E26^ns^	5±2E28^ns^	1±9E14^ns^	4±1E27^ns^	**–**	**–**
April 2008	0.37±0.17^ns^	−0.01±0.07^ns^	4.3	0.01	−2.5±5E21^ns^	12.5±1.E23^ns^	1.47±6E21^ns^	2.67±8E21^ns^	**–**	**–**
July 2008	0.29±0.14^ns^	0.02±0.05^ns^	4.58	0.04	11.25^ns^	7.5^a,ns^	8.58^a,ns^	2.667^ns^	**–**	**–**
October 2008	0.26±0.15^ns^	0.03±0.06^ns^	4.68	0.06	11.25±1E15^ns^	5±2E16^ns^	10±1E16^ns^	4±5E13^ns^	**–**	**–**

Significance levels for the parameters are shown by the same abbreviations as in Table1. See text for details on the meaning of the different parameters. Those parameter estimates for which no standard error values converged are shown by superscript (a).

**Table 5:** Relationship between log energy use and species-averaged log body size. We tested the fit of the linear and piecewise scaling models for all taxa. In those cases where parameter estimates did not converge, no parameter values are shown and are indicated with a dash symbol (–). The table shows the fitted values of the intercept (*β*_0_), slope of the first scaling region (*β*_1_), slope of the second scaling region (*β*_2_) and the threshold value (*τ*). Also shown are the Bayesian Information Criterion (BIC) and *R*^2^. Minimum values of BIC are highlighted in bold, indicating the selected model

Model	Linear				Piecewise					
Date	*β*_0_	*β*_1_	BIC	*R* ^2^	*β*_0_	*β*_1_	*β*_2_	*τ*	BIC	*R* ^2^
All taxa										
January 2007	1.06±0.69^ns^	0.23±0.24^ns^	**26.92**	0.15	**–**	**–**	**–**	**–**	**–**	**–**
May 2007	1.54±0.5^*^	0.18±0.21^ns^	**26.35**	0.13	1.06±0.85^ns^	−1.29±0.93^ns^	0.00±1.47^ns^	5±1.67^ns^	26.16	0.51
August 2007	1.48±0.69^ns^	0.2±0.23^ns^	24.64	0.16	2.92±1.06^ns^	−3.18±1.08^ns^	0.00±0.43^ns^	2.58±1.18^ns^	**15.89**	0.89
November 2007	1.66±0.56^*^	0.18±0.2^ns^	**31.98**	0.12	**–**	**–**	**–**	**–**	**–**	**–**
January 2008	2.05±0.35^*^^*^^*^	0.07±0.16^ns^	**46.68**	0.02	**–**	**–**	**–**	**–**	**–**	**–**
April 2008	2.63±0.52^*^	−0.04±0.22^ns^	**15.72**	0.01	**–**	**–**	**–**	**–**	**–**	**–**
July 2008	2.63±0.57^*^^*^	−0.12±0.2^ns^	**24.19**	0.06	−0.91±1.67^ns^	1.07±1.72^ns^	0.00±2.45^ns^	2.64±0.85^ns^	26.02	0.30
October 2008	2.52±0.72^*^	−0.1±0.28^ns^	**23.61**	0.03	−0.89±1.04^ns^	1.38±1.25^ns^	0.00±1.71^ns^	0±2.31^ns^	24.06	0.42

Significance levels for the slope parameters are shown by the same abbreviations as in Table1.

**Table 6:** Relationship between log species richness and log abundance within species-averaged body size classes. We tested the fit of the linear model for all taxa. The table shows the fitted values of the intercept (*β*_0_) and slope of the scaling region (*β*_1_), together with associated standard errors (see text for details on theoretical expectations). Also shown are the Bayesian Information Criterion (BIC) and *R*^2^

Model	Linear			
Date	*β*_0_	*β*_1_	BIC	*R* ^2^
All taxa				
January 2007	0.32±0.18^ns^	−0.03±0.07^ns^	8.47	0.05
May 2007	0.35±0.12^*^	0.01±0.05^ns^	5.77	0.00
August 2007	0.31±0.14^ns^	−0.01±0.05^ns^	5.62	0.01
November 2007	0.3±0.1^*^	0.02±0.04^ns^	4.52	0.03
January 2008	0.31^a,^^*^^*^^*^	0.01±^a,ns^	0.92	0.01
April 2008	0.37^a,ns^	−0.01±^a,ns^	0.00	0.01
July 2008	0.29±0.14^ns^	0.02±0.05^ns^	4.58	0.04
October 2008	0.26^a,ns^	0.03±^a,ns^	0.00	0.06

Significance levels for the slope parameters are shown by the same abbreviations as in Table1. Those parameter estimates for which no standard error values converged are shown by superscript (a).

**Figure 1:**
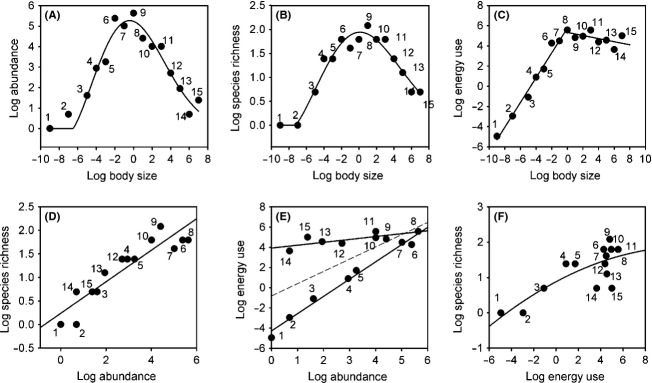
Bivariate relationships between individual-based log body size classes and log abundance, log species richness, and log energy use in the epibenthic faunal community of Coliumo Bay in January 2007. (A) Unimodal right-skewed relationship between body size and abundance. The line shows the Weibull fit to the individual-based data. (B) Unimodal right-skewed relationship between body size and species richness. The line shows the Weibull fit to the data. (C) Piecewise scaling relationship between body size and energy use. The line shows the fitted piecewise linear regression. (D) Scaling relationship between abundance and species richness. The line shows the fitted scaling relationship. (E) Scaling relationship between abundance and energy use. The dotted line shows the fitted linear regression for all the data, while continuous lines show the linear regressions fitted to data below and above the modal size class. (F) Bivariate relationship between energy use and species richness. The continuous line shows the second-order polynomial fitted to the data. In all figures, numbers refer to the body size classes.

**Figure 2:**
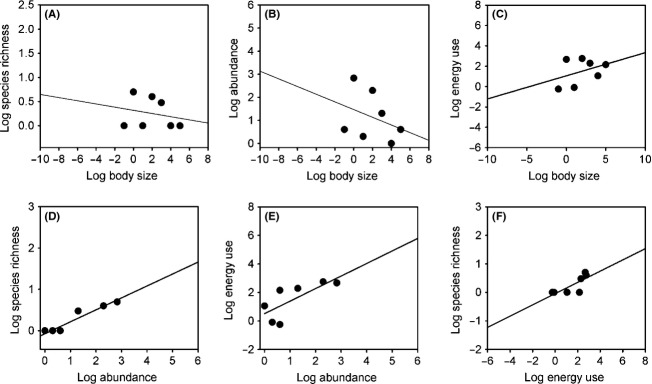
Bivariate relationships between species-averaged log body size, log species richness, log abundance and log energy use in the epibenthic faunal community of Coliumo Bay in January 2007. Lines shows the fitted linear regression for each relationships. (A) Relationship between average species body size and species richness. (B) Relationship between species-averaged body size and abundance. (C) Scaling relationship between body size and energy use. (D) Scaling relationship between abundance and species richness. (E) Scaling relationship between abundance and energy use. (F) Relationship between energy use and species richness.

**Figure 3:**
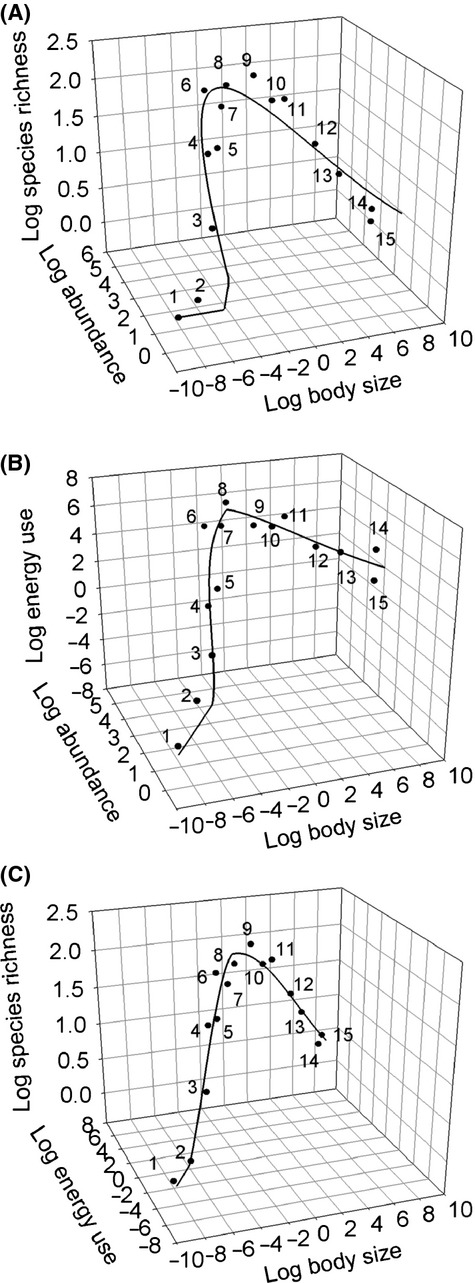
Trivariate relationships observed in the epibenthic faunal community of Coliumo Bay in January 2007. (A) The figure shows the relationship between log species richness and log abundance within log body size classes using fitted curves from Fig.A and 1B. (B) The figure shows relationship between log energy use and log abundance within log body size classes using fitted curves from Fig.A and 1C. (C) The figure shows relationship between log species richness and log energy use within log body size classes using fitted curves from Fig.B and C.

**Figure 4:**
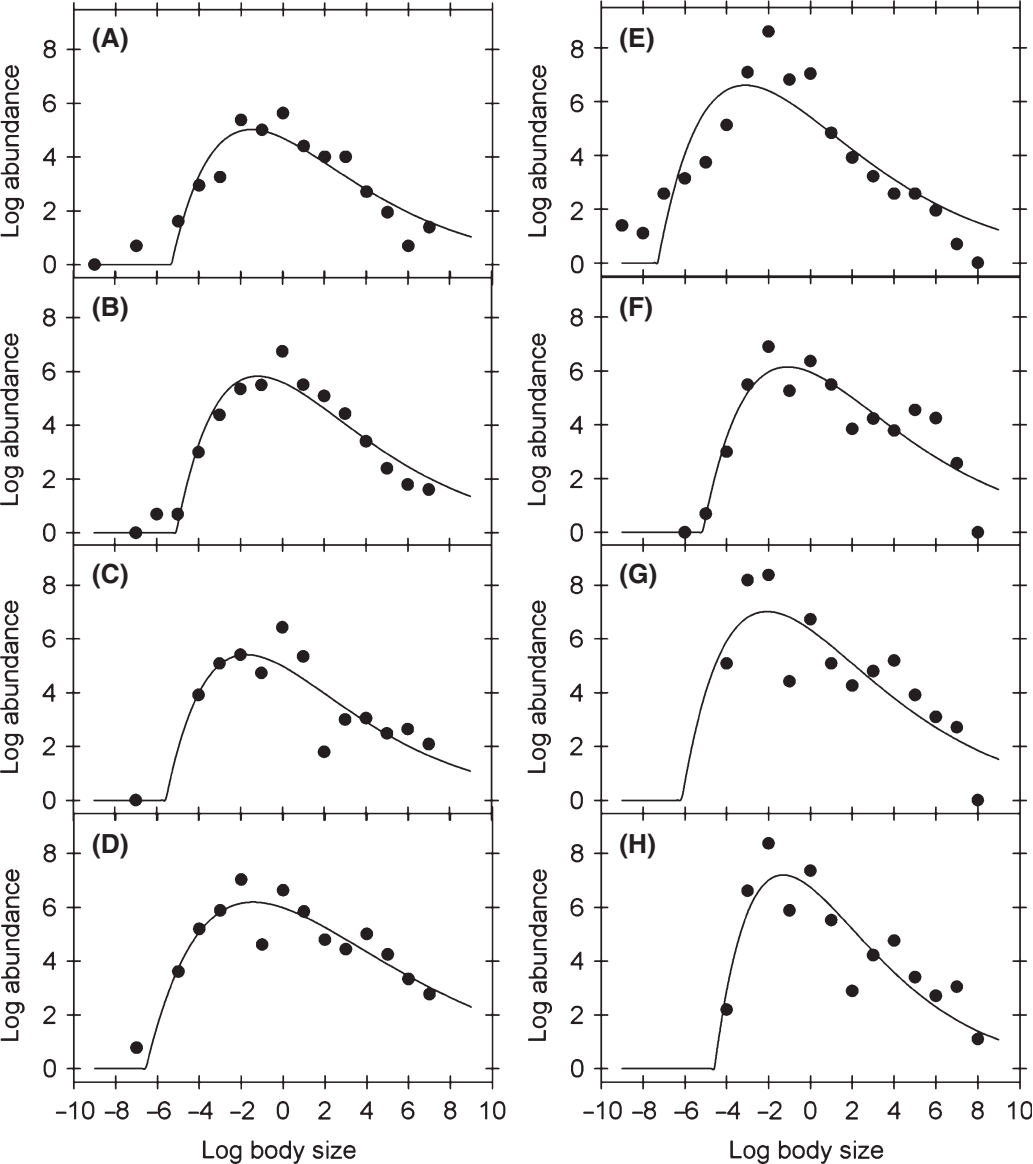
Temporal dynamics of the relationship between log abundance and log body size classes, fitted with log-Weibull functions. Figures A to D show the observed values and functions for January, May, August, and November 2007, respectively, while figures E to H show January, April, July, and October 2008, respectively. Parameter values and fitted *R*^2^ values are shown in TableA.

**Figure 5:**
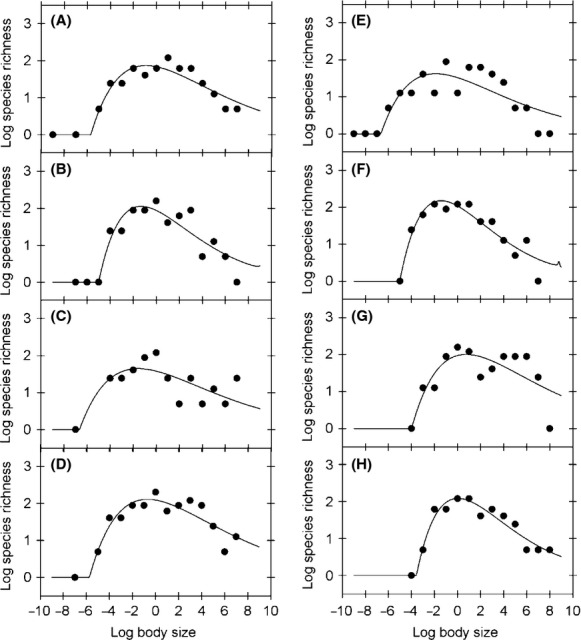
Temporal dynamics of the relationship between log species richness (S) and log body size classes, fitted with log-Weibull functions. Figures A to D show the observed values and functions for January, May, August, and November 2007, respectively, while figures E to H show January, April, July, and October 2008, respectively. Parameter values and fitted *R*^2^ values are shown in TableB.

Examination of the individual-based trivariate relationships observed in January 2007 shows that species richness and abundance followed a parabola across individual-based body size classes, whereas a less pronounced pattern is observed for the species richness–energy use relationship and the abundance–energy use relationship (Fig.3), which is likely due to the effect of the piecewise body size energy scaling (Fig.C). The SRA relationship over time revealed that abundance across individual-based body size classes is well explained by a unimodal right-skewed relationship in most of the samples examined, although deviations from unimodality seem apparent in some cases (Fig.4). The unimodal right-skewed pattern was more pronounced in January and November 2007 as well as in April and July 2008, as shown by the *R*^2^ values for the Weibull fit (Table1). Abundance also followed a right-skewed relationship between log abundance and log body size for most phyla and sampling dates, except for those arthropods sampled in the months of August and November 2007 and October 2008, where a linear model showed the lowest BIC values (see Table S2 in Appendix S1 and Figs S1 to S3 in Appendix S2). Contrary to expectations from the EER, we did not find support for a power law (i.e., significant linear fit) relationship between abundance and individual-based body size in any of the samples (Table1). A similar unimodal right-skewed pattern was observed for species richness and body size, with January and May 2007 and October 2008 showing higher *R*^2^ values for the Weibull relationship (Fig.5; Table1). Again, a power law relationship was not supported by the data in any of the samples examined (i.e., linear fit: Table1). Species richness, on the other hand, did not exhibit any significant unimodal pattern in any of the dominant phyla, except for Arthropoda which showed a significant Weibull relationship in the summer months of January 2007 and 2008 (see Table S3 in Appendix S1 and Figs S4–S6 in Appendix S2).

**Figure 6:**
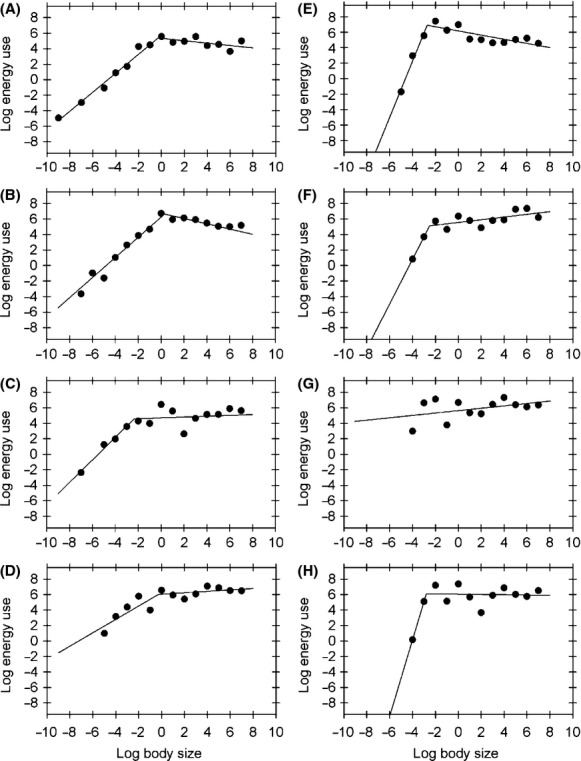
The figure shows temporal dynamics of the observed scaling of total log energy use (Watts) as a function of log body size classes. Filled circles show the total energy use in each body size class. Continuous lines show fitted piecewise linear regressions. Figures A to D show the observed values and fitted functions for January, May, August, and November 2007, respectively, while figures E to H show January, April, July, and October 2008, respectively. Parameter values and fitted *R*^2^ values are shown in Table2.

When we examined the scaling of total energy use across individual-based body size bins, we found that total energy use across all individuals could be well fit by a piecewise scaling relationship, rather than following a shallow, nonsignificant pattern as expected under the EER. In a similar pattern to that observed in January 2007, we found that in most samples, energy use increased up to a threshold body size, giving way to a very shallow or altogether flat scaling pattern for larger body sizes (Fig.6; Table2). The sole exception was the July 2008 sample, which exhibits a nonsignificant shallow positive slope (Table2). Thus, our data show that energetic equivalence is not a general feature of community structure for the epibenthic fauna of Coliumo Bay. Similar patterns were observed for Mollusca, such that scaling exponents for larger individuals did not differ from the energetic equivalence expectation (see Table S4 in Appendix S1, Fig. S7 in Appendix S2); while in Arthropoda, only the first 2 months showing a piecewise scaling pattern, and all remaining samples show a positive trend (see Table S4 in Appendix S1, Fig. S8 in Appendix S2). A positive linear trend was found for Chordata in most samples, with the exception of November 2007 (see Table S4 in Appendix S1, Fig. S9 in Appendix S2).

**Figure 7:**
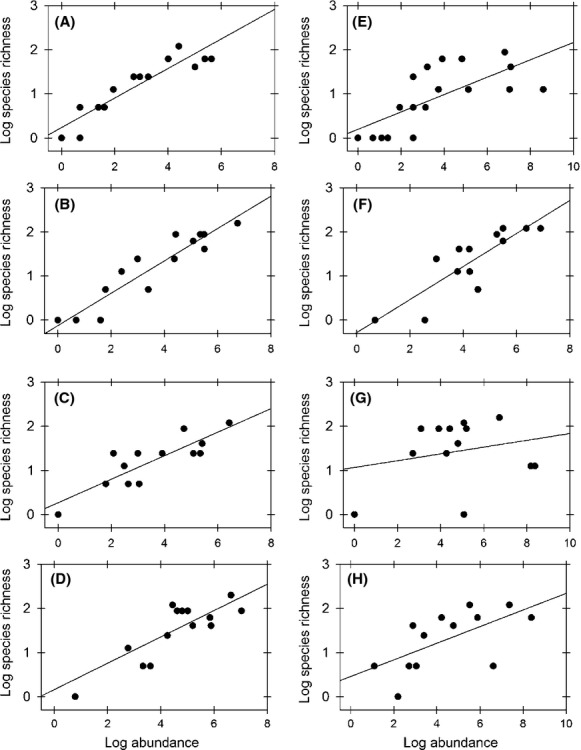
Temporal variation in the log species–log abundance curve. The figure shows the observed scaling of the log species richness as a function of the log-transformed abundance in body size classes. Filled circles show the species richness of each body size class. Continuous lines show fitted linear regressions. Figures A to D show the observed values and fitted OLS regressions for January, May, August, and November 2007, respectively, while figures E to H show January, April, July, and October 2008, respectively. Parameter values and fitted *R*^2^ values are shown in Table3.

Regarding species richness–abundance relationships, we found that with the exception of the July 2008 sample, all individual-based relationships were significant, with a variable goodness of fit; observed *R*^2^ ranged from 0.45 to 0.87 in all significant relationships (Table3). For the bivariate relationships between energy use and abundance, significant but weak linear scaling patterns are observed for all the samples, which provide a better explanation than piecewise scaling relationships for body size classes below and above the modal size class (Table3; Fig.6). Relationships between average species body size, species richness, and abundance follow power functions, with scaling exponents ranging between 0.14 and of 0.34 (Fig.7). For on major taxa (Mollusca, Arthropoda and, Chordata), no significant scaling was observed (see Table S5 in Appendix S1, Figs S16–S19 in Appendix S2).

For species-based trivariate SRA relationships across all the temporal samples studied, we found that abundance and species richness across the body size classes no longer follow a unimodal skewed pattern, presenting a linear pattern instead (Table4). When we examined these patterns within dominant phyla, in most cases, the lumping of data from individuals into a single body size value per species resulted in a reduction in the range of body size values, which in turn reduced the number of body size classes with observations. As a result, in many cases, there were insufficient data points to allow the fitting of a unimodal Weibull function, both in the full data set and within the dominant phyla (see Table S5 in Appendix S1, Figs S10–S15 in Appendix S2). Further, in many cases, the remaining data points do not present any significant trend.

For the energy scaling patterns across all taxa and within the dominant phyla, using species-averaged data, we found that in all cases, energy does not present any significant trend across body size classes (Table5). It must be noted that observed scaling exponent values for abundance across body size classes were not necessarily equal to the inverse of the exponent of the metabolic rate allometry (Nee et al. 1991). Nevertheless, energy use across body size classes did not show any significant trend (Table5, see Table S8 in Appendix S1, Figs S7–S9 in Appendix S2). Thus, species-based data do not falsify the null hypothesis corresponding to the EER expectation (Isaac et al. 2011). Finally, when we examine the species-based species richness–abundance relationships, we find no significant scaling relationships, both when all taxa were considered, nor when dominant phyla are considered. Exceptions to this latter trend were two samples in Mollusca and one sample in Arthropoda (see Table S6 in Appendix S1).

## Discussion

In the present study, we examined the empirical relationships among body size (S), species richness (R), and abundance (A) in a shallow epibenthic macro–megafaunal community. We also tested whether these data support energy equivalence by calculating individual energy use (E) across the same set of body size classes. When we examined individual-based data, we observe that both abundance and species richness across log body size classes followed a unimodal relationship in the samples examined, providing support for the generality of unimodal individual size spectra, as shown in grassland insects and gastropods (Siemann et al. 1996, 1999; Fa and Fa 2002; McClain 2004). On the other hand, examination of these relationships with species-based body size estimates results in a loss of the unimodal patterns. This may be due to the fact that the total number of species in this community at any given time does not exceed 20 species. Hence, averaging observed body sizes results in a strong decrease in the number of data points available to fit unimodal or linear relationships, and in most cases, no clear pattern is observed. Given that this decrease in the number of data points strongly constraints any attempt at modeling these patterns, we now focus on discussing the individual-based patterns. In this regard, a striking result is that although epibenthic faunal community composition and dominant species size structure in Coliumo Bay showed changes during the study period (Hernández-Miranda et al. 2010, 2012a,b), the overall pattern seems remarkably stable. While none of the individual-based samples provides evidence to support a power law scaling relationship of either species richness or abundance as a function of body size, there are noticeable deviations from the Weibull curve. On the other hand, species-based analyses show no significant pattern, either linear or Weibull, suggesting that the number of data points across size classes is not sufficient for any meaningful pattern to emerge. While these results add to the growing evidence for a unimodal SRA relationship in individual-based data, variation in the form of the relationship is observed. While data for January 2008 seem to show a relationship more similar to a log-normal, as has been reported for grassland insects (Siemann et al. 1996, 1999) and coastal gastropods (Fa and Fa 2002), all other samples show a right-skewed unimodal pattern similar to that reported for deep-sea gastropods (McClain 2004). It is interesting to note that the January 2008 sample was taken 4 days after a strong hypoxic disturbance that resulted in massive stranding of fish and marine invertebrates at Coliumo Bay (Hernández-Miranda et al. 2010, 2012a). Following this hypoxic disturbance, total community abundance increased, mostly due to the recruitment of scavenger snails species of the genus *Nassarius*, which apparently were favored trophically by the hypoxic mass mortality event (Hernández-Miranda et al. 2012a). The greater symmetry over a log scale for the community in this period likely reflects the greater representation of smaller individuals and the loss of the largest individuals, resulting in both a higher number of individuals and greater species richness in the lower size classes and greater symmetry. While further research would be necessary to demonstrate the causal role of recruitment dynamics and disturbance mortality on the shape of these frequency distributions, these results provide strong evidence of the modulating effects of the recruitment dynamics on the trivariate patterns examined.

Another variation we observed is the displacement of the body size distribution to the right in the austral winter and spring months. This may be partly related to the seasonal effects on reproductive patterns. The neritic and benthic communities inhabiting off Central Chile present a clear seasonal reproduction pattern, with the reproductive period occurring at the end of winter and/or the beginning of spring (Castro et al. 2000; Poulin et al. 2002; Hernández-Miranda et al. 2003). Following that, recruitment begins during spring season, promoted for the high primary productivity driven by coastal upwelling events (Palma et al. 2006; Pardo et al. 2007; Sobarzo et al. 2007). Hence, the frequency of smaller individuals during spring may be explained by the arrival of new individuals or recruits. On the other hand, the increased frequency of larger individuals may reflect the arrival of larger, more mobile organisms such as rays or the toadfish *Aphos porosus* (Hernández-Miranda et al. 2012b). It may be argued that our analysis of species-based data does not reflect such changes in body size frequency. However, it is important to note that in these samples, there is a strong degree of heterogeneity or skewness in the size of observed individuals, particularly of those undergoing recruitment or migration events following disturbances such as hypoxia. This variability is not considered when data are averaged at the level of species.

Regarding the species–abundance scaling relationship, we found that with the exception of the July 2008, the observed scaling exponent was statistically significant and did not differ from 0.5 (Table3), while no significant scaling emerged in species-based data. Thus, the majority of our results are in agreement with previous individual-based studies (McClain 2004), as well as with results in species-based studies (Siemann et al. 1996, 1999; Fa and Fa 2002). It has been shown that within a community, *b* equals 0.5 when relative abundances follow a broken-stick model or equals 0.25 when relative abundances follow a canonical log-normal distribution (May 1988). As discussed earlier, with the sole exception of the observed community after hypoxia in January 2008, body size distributions are right-skewed rather than log-normal. Thus, the observed pattern may be explained by the apportionment of resources among epibenthic faunal species in this community, which would be coherent with an individual rather than species-based view.

This highlights the fact that body size is likely a latent or underlying variable in the relationship between energy use and abundance. However, energy use per log body size class increased monotonically up to the modal class, with no statistical differences for sizes larger than or equal to the modal size class. Although a unimodal abundance distribution would suggest that EER does not hold true for this community, the piecewise scaling of energy use shows it may be a valid generalization for larger individuals inhabiting Coliumo Bay. The importance of the modal size class is highlighted by the fact that the fitted piecewise thresholds *τ* in the energy scaling relationship are strongly correlated with the fitted Weibull location parameter *θ* for abundance (Pearson correlation = 0.83). It is important to note that the fitted value of *θ* corresponds to the estimated modal body size for the Weibull function. Thus, contrary to expectations from previous studies on allometric scaling, EER holds only for body sizes larger than the modal size class (Table3). It is important to remark that our results provide evidence for a piecewise energy scaling based on energy use estimates obtained for every individual, rather than assuming average body size and an expected population energy use; hence, our result does not rely on assumptions regarding the average body size of a species. Also, given that energy use was estimated using known allometric relationships for each taxon, these results do not assume universality of scaling exponents. The results also show that unimodal size spectra may yield a different outcome than the assumed EER relationship. This may provide interesting ground to examine possible ways to generalize and extend the MTE.

One important point remains as to whether species or individual-based distributions should be examined. While initial studies of the SRA relationship focused on species-based patterns, latter examples showed that the trivariate SRA relationship could hold true for individual-based data. Our results show that in relatively species-poor communities, the trivariate SRA may be present in individual-based data, but not necessarily when species-based data are considered. While there is a long tradition of studies examining biomass spectra or individual size spectra, where the phenomena are analyzed using data at the level of the individual, most macroecological patterns have been examined under the assumption that body size is a species-level trait. In this regard, while there has been recent discussion regarding whether neutral models are relevant to understand community dynamics, there has been less emphasis on whether individual- or species-level phenomena shape patterns in community structure.

Macroecological research has often sought to identify statistical regularities by examining relationships among variables such as body size, abundance, geographic range, and species richness. For the most part, these patterns have been studied by focusing on either univariate or bivariate relationships (Gaston and Blackburn 2000; Blackburn and Gaston 2003). Thus macroecology has been viewed by some as a statistical extension of biogeography (but see Marquet 2002; Blackburn and Gaston 2002). On the other hand, studies on the dynamics of macroecological relationships have by and large been scarce (but see Keitt et al. 2002). Most macroecological studies have emphasized testing the generality of scaling or invariant relationships and their derived predictions, rather than the specific dynamics of particular systems (e.g., Brown et al. 2004). In this regard, it may seem that macroecological patterns cannot provide insights into the patterns of community dynamics. However, it has been suggested that ecological scaling relationships may serve as baselines or attractors describing the steady-state structure and functioning of ecological systems (Kerkhoff and Enquist 2007). If this is the case, departures from the expected scaling relationship may serve as signatures or indicators of the disproportionate influence of particular processes structuring the community or ecosystem (Kerkhoff and Enquist 2007). This line of reasoning has led to the proposition that allometrically derived size distributions are asymptotic attractors, and disturbances generate systematic deviations from the expected steady-state structure. This perspective suggests a mechanistic link between the dynamics of ecosystems and their scaling properties. In this contribution, we have re-examined the SRA relationship and broadened its scope by including an explicit estimate of individual energy use. This allowed us to examine abundance, richness and energy use across all species found in a given size class. Hence, we examined how much energy is partitioned as a function of an individual's body size irrespective of its taxonomic classification. The individual-based perspective has shown the SRA relationship to be consistent with previously documented patterns, despite methodological differences across some of these studies. Determining the actual causes of temporal variations and deviations from the unimodal relationships requires further research, in order to ascertain potential effects from sampling or noise artifacts. In this regard, it is remarkable that the piecewise energy use scaling observed here shows that unimodal biomass distributions deviate from the EER expectation for individuals of small body sizes. Examination of species-based data for this community obscured the observed SRA patterns, which we think may be due to the decrease in the number of degrees of freedom as a result of species averaging. If this is the case, these results may provide a potential reason for the lack of evidence supporting energetic equivalence at the local scales. Finally, future work should examine the generality of unimodal SRA relationships and piecewise energy use scaling in other taxa and ecological systems.

## Supporting Information

**Table S1** List of epibenthic macro and megafaunal resident species observed at Coliumo Bay during the time series (2007-2008). **Table S2** Relationship between log abundance and individual-based log body size. **Table S3** Relationship between log species richness and individual-based log body size. **Table S4** Relationship between log energy use and individual-based log body size. **Table S5** Relationship between log species richness and log abundance within individual based body size classes. **Table S6** Relationship between log abundance and log species-averaged body size data. **Table S7** Relationship between log species richness and log species-averaged body size data. **Table S8** Relationship between log energy use and species-averaged log body size. **Table S9** Relationship between log species richness and log abundance within species - averaged body size classes. **Figure S1** Temporal dynamics in the relationship between log abundance and log individual-based body size classes in Phylum Mollusca. **Figure S2** Temporal dynamics in the relationship between log abundance and log individual-based body size classes in Phylum Arthropoda. **Figure S4** Temporal dynamics in the relationship between log species richness and individual based body size classes in Phylum Mollusca. **Figure S5** Temporal dynamics in the relationship between log species richness and log individual based body size classes in Phylum Arthropoda. **Figure S6** Temporal dynamics in the relationship between log species richness and log individual based body size classes in Phylum Chordata. **Figure S7** The figure shows temporal dynamics of the observed scaling of log total energy use (Watts) as a function of log individual based body size classes in Phylum Mollusca. **Figure S8** The figure shows temporal dynamics of the observed scaling of log total energy use (Watts) as a function of log individual based body size classes in Phylum Arthropoda. **Figure S9** The figure shows temporal dynamics of the observed scaling of log total energy use (Watts) as a function of log individual based body size classes in Phylum Chordata. **Figure S10** Temporal dynamics in the relationship between log abundance and species averaged log body size classes in Phylum Mollusca. **Figure S11** Temporal dynamics in the relationship between log abundance and species averaged log body size classes in Phylum Arthropoda. **Figure S12** Temporal dynamics in the relationship between log abundance and species averaged log body size classes in Phylum Chordata. **Figure S13** Temporal dynamics in the relationship between log species richness and species averaged log body size classes in Phylum Mollusca. **Figure S14** Temporal dynamics in the relationship between log species richness and species averaged log body size classes in Phylum Arthropoda. **Figure S15** Temporal dynamics in the relationship between log species richness and species averaged log body size classes in Phylum Chordata. **Figure S16** The figure shows temporal dynamics of the observed scaling of log total energy use (Watts) as a function of species averaged log body size classes in Phylum Mollusca. **Figure S17** The figure shows temporal dynamics of the observed scaling of log total energy use (Watts) as a function of species averaged log body size classes in Phylum Arthropoda. **Figure S18** The figure shows temporal dynamics of the observed scaling of log total energy use (Watts) as a function of species averaged log body size classes in phylum Chordata.
