# Supplementary material for: Dynamic relationships between body size, species richness, abundance, and energy use in a shallow marine epibenthic faunal community
Source: Ecol Evol. 2014 Dec 28;5(2):391–408. doi: 10.1002/ece3.1343 (PMC4314271; doi:10.1002/ece3.1343)
Supplement: Supplementary file 1 [file ece30005-0391-sd1.docx]

Supporting Information

**Dynamic relationships between body size, species richness, abundance and energy use in a shallow marine epibenthic faunal community**

**Fabio A. Labra^1^, Eduardo Hernández-Miranda^2,3*^ & Renato A. Quiñones^2,3^**

^1^Facultad de Ciencias, Universidad Santo Tomas. Ejercito 146, Código Postal 8370003, Santiago, Chile.

^2^Programa de Investigación Marina de Excelencia (PIMEX), Facultad de Ciencias Naturales & Oceanográficas, Universidad de Concepción.

^3^Interdisciplinary Center for Aquaculture Research (INCAR), Casilla 160-C, Universidad de Concepción, Concepción, Chile.

Authors’ email: flabra@santotomas.cl, eduhernandez@udec.cl, rquinone@udec.cl

**Keywords**: Macroecological Dynamics, Energetic Equivalence, South Eastern Pacific, Hypoxia, Coliumo Bay, Humboldt Current System.

Short running-title: “**Body size, species richness, abundance and energy use in a marine community**”

*corresponding author: Eduardo Hernández-Miranda, eduhernandez@udec.cl

**Appendix S1:**

**Supporting Tables**

**Table S1**. List of epibenthic macro and megafaunal resident species observed at Coliumo Bay during the time series (2007-2008). Feeding guild were assigned according to Gutiérrez et al. (2000) and Laudien et al. (2007). The feeding guilds of species not described by these authors were assigned based on the phylogenetically closest described species. C = Carnivorous, O = Omnivorous.

| **Phylum** | **Class** | **Order** | **Family** | **Species** | **Feeding guild** |
| --- | --- | --- | --- | --- | --- |
| Equinodermata | Asteroidea | Valvatida | Asterinidae | *Patiria chilensis* (Verrill, 1870) | C |
|  |  | Forcipulatida | Stichasteridae | *Stichaster striatus* (Müller & Troschel, 1840) | C |
|  |  |  |  |  |  |
| Chordata | Elasmobranchii | Carcharhiniformes | Scyliorhinidae | *Schroederichthys chilensis* (Guichenot, 1848) | C |
|  |  | Rajiformes | Rajidae | *Zearaja chilensis* (Guichenot, 1848) | C |
|  | Actinopterygii | Batrachoidiformes | Batrachoididae | *Aphos porosus* (Valenciennes, 1837) | C |
|  |  | Gadiformes | Merluccidae | *Merluccius gayi* (Guichenot, 1848) | C |
|  |  | Ophidiiformes | Ophidiidae | *Genypterus maculatus* (Tschudi, 1846) | C |
|  |  | Perciformes | Mugilidae | *Mugiloides chilensis* (Molina, 1782) | C |
|  |  |  | Pinguipedidae | *Prolatilus jugularis* (Valenciennes, 183 | C |
|  |  | Pleuronectiformes | Paralichthydae | *Paralichthys adspersus* (Steindachner, 1867) | C |
|  |  | Scorpaeniformes | Agonidae | *Agonopsis chiloensis* (Jenyns, 1840) | C |
|  |  |  | Congiopodidae | *Congiopodus peruvianus* (Cuvier, 1829) | C |
|  |  |  | Normanichthyidae | *Normanichthys crockeri* (Clark, 1937) | C |
|  |  |  | Sebastidae | *Sebastes oculatus* (Valenciennes, 1833) | C |
|  | |  |  |  |  |
| Arthropoda | Malacostraca | Decapoda | Alpheidae | *Betaeus truncatus* (Dana, 1852) | C |
|  |  |  | Calapidae | *Mursia gaudichaudi* (H. Milne Edwards, 1834) | C |
|  |  |  | Callianassidae | *Callianassa garthi* (Retamal, 1975) | C |
|  |  |  | Cancridae | *Cancer coronatus* (Molina, 1782) | C |
|  |  |  |  | *Metacarcinus edwardsii* (Bell, 1835) | C |
|  |  |  |  | *Cancer porteri* (Rathbun, 1930) | C |
|  |  |  |  | *Cancer setosus* (Molina, 178) | C |
|  |  |  | Atelecyclidae | *Pseudocorystes sicarius* (Poeppig, 1836) | C |
|  |  |  | Inachidae | *Eurypodius latreillii* (Guérin, 1825) | O |
|  |  |  | Epialtidae | *Taliepus dentatus* (H. Milne Edwards, 1834) | O |
|  |  |  | Munididae | *Pleuroncodes monodon* (H. Milne Edwards, 1837) | C |
|  |  |  | Pandalidae | *Heterocarpus reedi* (Bahamonde N., 1955) | C |
|  |  |  | Paguridae | *Pagurus perlatus* (H. Milne Edwards, 1848) | C |
|  |  |  | Paguridae | *Pagurus villosus* (Nicolet, 1849) | C |
|  |  |  | Pinnotheridae | *Pinnixa bahamondei* (Garth, 1957) | O |
|  |  |  | Polybiidae | *Ovalipes trimaculatus* (De Haan, 183) | C |
|  |  |  | Rhynchocinetidae | *Rhynchocinetes typus* (H. Milne Edwards, 1837b) | C |
|  |  |  | Platyxanthidae | *Homalaspis plana* (H. Milne Edwards, 1834) | C |
|  |  |  |  |  |  |
| Mollusca | Gastropoda | Archaeogastropoda | Turbinidae | *Tegula atra* (Lesson, 1830) | C |
|  |  | Neogastropoda | Muricidae | *Chorus giganteus* (Lesson, 1831 | C |
|  |  |  |  | *Trophon geversianus* (Pallas, 1774) | C |
|  |  |  |  | *Xanthochorus cassidiformis* (Blainville, 1832) | C |
|  |  |  |  | *Crassilabrum crassilabrum* (Sowerby, 1834) | C |
|  |  |  | Nassariidae | *Nassarius coppingeri* (Smith 1881) | C |
|  |  |  |  | *Nassarius dentifer* (Powys, 1835) | C |
|  |  |  |  |  |  |

**Table S2**: Relationship between log abundance and individual-based log body size. The table shows the estimated values for the 4-parameter Weibull function and the linear (power law) fit for individual based body size. Analysis are shown for the three dominant Phyla: Mollusca, Arthropoda and Chordata. Data for Equinodermata did not allow sufficient sample size for statistical testing and hence are not shown. Also shown are the Bayesian Information Criterion (BIC) and R^2^ values for each model. Minimum values of BIC are highlighted in bold, indicating the selected model. Significance results for the slope parameters are shown by the following abbreviations: ***: p<0.001; **: p<0.01; *: p<0.05; ns: p>0.05. See text for details on the meaning of the different parameters.

| Model | Linear |  | |  | |  | Weibull |  |  |  |  | | |  | |
| --- | --- | --- | --- | --- | --- | --- | --- | --- | --- | --- | --- | --- | --- | --- | --- |
| Date | β_0_ | β_1_ | | BIC | | R^2^ | θ | α | γ | κ | BIC | | | R^2^ | |
| **Mollusca** |  | |  | |  |  |  |  |  |  | |  |  | |  |
| January 2007 | 2.37±0.79* | | 0.19±0.24^ns^ | | 21.21 | 0.08 | 1.51±0.57* | 5.82±0.72*** | 3.24±0.71** | 1.67±0.59* | | **8.22** | 0.79 | |  |
| May 2007 | 5.62±1.31* | | -0.68±0.37^ns^ | | 12.93 | 0.33 | 2.28±0.42* | 6.18±0.56** | 33.74±258^ns^ | 19.1±141.64^ns^ | | **3.57** | 0.90 | |  |
| August 2007 | 6.26±1.36* | | -0.9±0.38^ns^ | | 13.79 | 0.49 | 1.87±0.41* | 6.74±1.24* | 7E05±2E08^ns^ | 4E04±1E08^ns^ | | **11.1** | 0.70 | |  |
| November 2007 | 7.2±1.2** | | -1.18±0.31* | | 14.45 | 0.69 | 1.46±0.75^ns^ | 7.23±0.85** | 6E04±3E07^ns^ | 3E04±1E08^ns^ | | **8.82** | 0.88 | |  |
| January 2008 | 4.31±0.96** | | 0±0.3^ns^ | | 28.33 | 0 | 1.32±0.19*** | 8.04±0.35^ns^ | 12.4±5.82^ns^ | 5.69±2.69^ns^ | | **-1.33** | 0.96 | |  |
| April 2008 | 7.01±1.04** | | -0.99±0.27* | | 11.08 | 0.71 | 1.72±0.36* | 6.7±0.36** | 3E05±4E07^ns^ | 1E05±2E07^ns^ | | **0.17** | 0.97 | |  |
| July 2008 | 8.89±0.34^ns^ | | -1.22±0.1*** | | **-0.43** | 0.97 | 0.44±0.08* | 8.66±0.31** | 3.32±0.26** | 1.26±0.15* | | -8.4 | 0.99 | |  |
| October 2008 | 8.64±0.97*** | | -1.21±0.27* | | 9.95 | 0.79 | 1.22±0.18* | 8.21±0.38** | 4.32±1.7^ns^ | 2.15±0.77^ns^ | | **-1.73** | 0.98 | |  |
|  |  | |  | |  |  |  |  |  |  | |  |  | |  |
| **Arthropoda** |  | |  | |  |  |  |  |  |  | |  |  | |  |
| January 2007 | 2.35±0.4*** | | 0.07±0.13^ns^ | | 13.02 | 0.03 | 1.11±0.58^ns^ | 3.77±0.42*** | 23.29±63.58^ns^ | 9.21±24.69^ns^ | | **3.05** | 0.64 | |  |
| May 2007 | 2.97±0.55*** | | 0.28±0.17^ns^ | | 21.17 | 0.13 | 0.58±0.26^ns^ | 5.42±0.29^ns^ | 5.56±0.47^ns^ | 2.09±0.31*** | | **-4.69** | 0.93 | |  |
| August 2007 | 3.2±0.58** | | -0.2±0.21^ns^ | | **12.18** | 0.13 | -59±9E07^ns^ | 416±3E09^ns^ | 12±7E04^ns^ | 1±2E03^ns^ | | 14.8 | 0.16 | |  |
| November 2007 | 4.48±0.24^ns^ | | -0.09±0.09^ns^ | | **2.04** | 0 | 0.42±1.53^ns^ | 4.74±0.41^ns^ | 2E05±2E08^ns^ | 2E04±1E07^ns^ | | 3.07 | 0.29 | |  |
| January 2008 | 2.19±0.42*** | | -0.05±0.13^ns^ | | 14.82 | 0 | 1.48±1.26^ns^ | 3.33±0.53*** | 1E04±4E07^ns^ | 5E03±1E07^ns^ | | **8.46** | 0.42 | |  |
| April 2008 | 3.56±0.45^ns^ | | 0.24±0.16^ns^ | | 11.84 | 0.13 | 0.94±0.47^ns^ | 4.74±0.3^ns^ | 8.38±1.92** | 1.49±0.18*** | | **-1.6** | 0.84 | |  |
| July 2008 | 3.31±0.74** | | 0.18±0.26^ns^ | | 11.12 | 0.09 | 2.08±0.37* | 5.05±0.39** | 5.31±1.02* | 2.18±0.75^ns^ | | **0.95** | 0.8 | |  |
| October 2008 | 2.98±0.51** | | 0.14±0.18^ns^ | | **6.55** | 0.11 | 2.15±13.3^ns^ | 5.78±42.07^ns^ | 3.09±16.43^ns^ | 1.04±4^ns^ | | 18.98 | 0 | |  |
|  |  | |  | |  |  |  |  |  |  | |  |  | |  |
| **Chordata** |  | |  | |  |  |  |  |  |  | |  |  | |  |
| January 2007 | 1.03±0.5^ns^ | | -0.02±0.22^ns^ | | 5.65 | 0 | 2.74±0.01^ns^ | 5.83±2E08^ns^ | 0.34±1E07^ns^ | 2.15±4E07^ns^ | | **11.69** | 0 | |  |
| May 2007 | 3.01±0.65* | | -0.85±0.46^ns^ | | 8.55 | 0.37 | -0.86±0.5^ns^ | 5.21±0.95^ns^ | 3.24±4.91^ns^ | 2.78±4.1^ns^ | | **5.75** | 0.69 | |  |
| August 2007 | 1.32±0.57^ns^ | | -0.21±0.23^ns^ | | 9.08 | 0 | -0.91±1.68^ns^ | 5.38±21.15^ns^ | 1.03±1.03^ns^ | 1.08±1.34^ns^ | | **-4.82** | 0.94 | |  |
| November 2007 | 3.16±0.78* | | -0.08±0.35^ns^ | | 14.59 | 0 | 0.33±0.21^ns^ | 5.11±0.33*** | 3.68±0.25*** | 1.92±0.24** | | **-1.67** | 0.92 | |  |
| January 2008 | 0.3±0.55^ns^ | | 0.3±0.29^ns^ | | 2.16 | 0.01 | 1.82^ns^ | 1.65±0*** | 1.57±0*** | 1.95±0** | | **-1.67** | 0.92 | |  |
| April 2008 | 2.86±0.27*** | | -0.74±0.13** | | 1.61 | 0.87 | -1.65±2E04^ns^ | 5.08±29447.64^ns^ | 2.42±36.29^ns^ | 1±14.3^ns^ | | **18.3** | 0 | |  |
| July 2008 | 1.39±0.58^ns^ | | 0.01±0.19^ns^ | | 7.7 | 0 | -2±2E04^ns^ | 4.47±2E04^ns^ | 4.26±30.41^ns^ | 1±6.4^ns^ | | **-2.77** | 0.85 | |  |
| October 2008 | 1.73±0.59* | | -0.08±0.18^ns^ | | 13.77 | 0 | -0.74±2.69^ns^ | 3.79±4.54^ns^ | 3.36±1.91ns | 1.07±0.71^ns^ | | **9.82** | 0.32 | |  |

**Table S3**: Relationship between log species richness and individual-based log body size. The table shows the estimated values for the 4-parameter Weibull function and the linear (power law) fit. Analysis are shown for the three dominant phyla: Mollusca, Arthropoda and Chordata. In those cases where parameter estimates did not converge, no parameter values are shown and are indicated with a dash symbol (-). Data for Equinodermata did not allow sufficient sample size for statistical testing and hence are not shown. Also shown are the Bayesian Information Criterion (BIC) and R^2^ values for each model. Minimum values of BIC are highlighted in bold, indicating the selected model. Significance results for the slope parameters are shown by the same abbreviations as in Table S2. See text for details on the meaning of the different parameters.

| Model | Linear |  | |  | | |  | Weibull | |  | |  | |  |  |  | |
| --- | --- | --- | --- | --- | --- | --- | --- | --- | --- | --- | --- | --- | --- | --- | --- | --- | --- |
| Date | β_0_ | β_1_ | | BIC | | | R^2^ | θ | | α | | γ | | κ | BIC | R^2^ | |
| **Mollusca** |  | |  | |  |  | | |  | |  | |  |  |  | |  |
| January 2007 | 0.12±0.14^ns^ | 0±0.04^ns^ | | **12.99** | | | 0 | - | | - | | - | | - | - | - | |
| May 2007 | 0.34±0.19^ns^ | 0.01±0.08^ns^ | | **9.65** | | | 0.01 | - | | - | | - | | - | - | - | |
| August 2007 | 0.18±0.22^ns^ | 0.01±0.09^ns^ | | **11.68** | | | 0 | - | | - | | - | | - | - | - | |
| November 2007 | 0.58±0.19* | -0.15±0.07^ns^ | | **11.77** | | | 0.44 | - | | - | | - | | - | - | - | |
| January 2008 | 0.12±0.14 ^ns^ | 0±0.04 ^ns^ | | **19.05** | | | 0 | - | | - | | - | | - | - | - | |
| April 2008 | 0.34±0.19 ^ns^ | 0.01±0.08 ^ns^ | | **11.77** | | | 0.01 | - | | - | | - | | - | - | - | |
| July 2008 | 0.18±0.22 ^ns^ | 0.01±0.09 ^ns^ | | **10.35** | | | 0 | - | | - | | - | | - | - | - | |
| October 2008 | 0.58±0.19* | -0.15±0.07 ^ns^ | | **19.05** | | | 0.44 | - | | - | | - | | - | - | - | |
|  |  |  | |  | | |  |  | |  | |  | |  |  |  | |
| **Arthopoda** |  |  | |  | | |  |  | |  | |  | |  |  |  | |
| January 2007 | 0.4±0.14* | 0.08±0.04 ^ns^ | | 16.03 | | | 0.28 | 1.4±0.71 ^ns^ | | 0.97±0.2** | | 3.98±0.93** | | 1.72±0.48* | **-13.4** | 0.62 | |
| May 2007 | 0.35±0.17 ^ns^ | 0.08±0.05 ^ns^ | | **24.04** | | | 0.19 | - | | - | | - | | - | - | - | |
| August 2007 | 0.66±0.23* | -0.06±0.09 ^ns^ | | **17.14** | | | 0.07 | - | | - | | - | | - | - | - | |
| November 2007 | 0.4±0.2 ^ns^ | 0.12±0.07 ^ns^ | | **19.37** | | | 0.3 | - | | - | | - | | - | - | - | |
| January 2008 | 0.4±0.14* | 0.08±0.04 ^ns^ | | 19.94 | | | 0.28 | 0.74±0.43 ^ns^ | | 1.05±0.14*** | | 4.14±0.46*** | | 1.87±0.31*** | **-21.54** | 0.78 | |
| April 2008 | 0.35±0.17 ^ns^ | 0.08±0.05 ^ns^ | | **19.37** | | | 0.19 | - | | - | | - | | - | - | - | |
| July 2008 | 0.66±0.23* | -0.06±0.09 ^ns^ | | **15.81** | | | 0.07 | - | | - | | - | | - | - | - | |
| October 2008 | 0.4±0.2 ^ns^ | 0.12±0.07 ^ns^ | | **19.94** | | | 0.3 | - | | - | | - | | - | - | - | |
|  |  |  | |  | | |  |  | |  | |  | |  |  |  | |
| **Chordata** |  |  | |  | | |  |  | |  | |  | |  |  |  | |
| January 2007 | 0.29±0.21 ^ns^ | -0.04±0.09 ^ns^ | | **8.72** | | | 0.04 | - | | - | | - | | - | - | - | |
| May 2007 | 0.28±0.16 ^ns^ | 0.14±0.11 ^ns^ | | **6.19** | | | 0.33 | - | | - | | - | | - | - | - | |
| August 2007 | 0.13±0.14 ^ns^ | -0.02±0.06 ^ns^ | | **6** | | | 0.03 | - | | - | | - | | - | - | - | |
| November 2007 | 0.41±0.2 ^ns^ | 0.23±0.09 ^ns^ | | **12.91** | | | 0.57 | - | | - | | - | | - | - | - | |
| January 2008 | 0.29±0.21 ^ns^ | -0.04±0.09 ^ns^ | | **6.09** | | | 0.04 | - | | - | | - | | - | - | - | |
| April 2008 | 0.28±0.16 ^ns^ | 0.14±0.11 ^ns^ | | **12.91** | | | 0.33 | - | | - | | - | | - | - | - | |
| July 2008 | 0.13±0.14 ^ns^ | -0.02±0.06 ^ns^ | | **14.47** | | | 0.03 | - | | - | | - | | - | - | - | |
| October 2008 | 0.41±0.2 ^ns^ | 0.23±0.09 ^ns^ | | **6.09** | | | 0.57 | - | | - | | - | | - | - | - | |

**Table S4**: Relationship between log energy use and individual-based log body size. We tested the fit of the linear and piecewise scaling models for the three dominant Phyla: Mollusca, Arthropoda and Chordata. In those cases where parameter estimates did not converge, no parameter values are shown and are indicated with a dash symbol (-). Data for Equinodermata did not allow sufficient sample size for statistical testing and hence are not shown. The table shows the fitted values of the intercept (β_0_), slope of the first scaling region (β_1_), slope of the second scaling region (β_2_) and the threshold value (τ). Also shown are the Bayesian Information Criterion (BIC) and R^2^. Minimum values of BIC are highlighted in bold, indicating the selected model. Significance levels for the slope parameters are shown by the same abbreviations as in Table S2.

| Model | Linear: Y= β_0_+ β_1_X | | | | Piecewise: Y= β_0_+ β_1_X - β_2_(X- τ)+ | | | | | |
| --- | --- | --- | --- | --- | --- | --- | --- | --- | --- | --- |
| Date | β_0_ | β_1_ | BIC | R^2^ | β_0_ | β_1_ | β_2_ | τ | BIC | R^2^ |
| **Mollusca** |  |  |  |  |  |  |  |  |  |  |
| January 2007 | 2.89±0.77** | 1.17±0.24** | 44.26 | 0.77 | 5.85±0.67*** | 2.1±0.19*** | -2.32±0.29*** | 0±0.38^ns^ | **25.17** | 0.98 |
| May 2007 | 4.45±0.82** | 0.2±0.36^ns^ | 27.4 | 0.07 | 6.46±1.07* | 2.19±0.83^ns^ | -2.81±0.99 ^ns^ | 0±0.68^ns^ | **21.29** | 0.82 |
| August 2007 | 4.68±0.83** | 0.08±0.35^ns^ | **27.77** | 0.01 | 5.77±5.24^ns^ | 1.23±3.32^ns^ | -1.51±3.37 ^ns^ | 0±3.6^ns^ | 29.62 | 0.26 |
| November 2007 | 5.01±0.71*** | -0.21±0.28^ns^ | **30.6** | 0.1 | - | - | - | - | - | - |
| January 2008 | 4.42±1** | 0.97±0.29* | 54.6 | 0.58 | 9.87±0.52*** | 2.45±0.13*** | -3.14±0.22*** | 0±0.23^ns^ | **23.29** | 0.99 |
| April 2008 | 5.2±0.63** | -0.04±0.24^ns^ | 24.16 | 0.01 | 8.24±1.89* | 2.4±1.2^ns^ | -2.85±1.22^ns^ | 0±0.5^ns^ | **17.38** | 0.82 |
| July 2008 | 6.59±0.17*** | -0.25±0.07* | **8.28** | 0.74 | - | - | - | - | - | - |
| October 2008 | 6.37±0.57*** | -0.27±0.25^ns^ | 23.14 | 0.23 | 9.65±0.2*** | 2.28±0.13** | -3.06±0.13** | 0±0.05^ns^ | **-9.49** | 1 |
|  |  |  |  |  |  |  |  |  |  |  |
| **Arthropoda** |  |  |  |  |  |  |  |  |  |  |
| January 2007 | 2.52±0.42*** | 1.01±0.14*** | 38.69 | 0.87 | 3.51±0.4*** | 1.49±0.16*** | -1.45±0.37** | 0±0.67^ns^ | **29.19** | 0.97 |
| May 2007 | 3.1±0.55*** | 1.25±0.17*** | 49.4 | 0.85 | 5.26±0.41*** | 2.09±0.14*** | -2.07±0.23*** | 0±0.34^ns^ | **25.99** | 0.99 |
| August 2007 | 3.22±0.65** | 0.82±0.24* | 33.65 | 0.66 | 2.63±1.28^ns^ | -0.14±0.99^ns^ | 1.6±1.08^ns^ | 0±1.49^ns^ | **32.91** | 0.82 |
| November 2007 | 4.66±0.26*** | 0.89±0.09*** | 23.93 | 0.93 | 4.77±0.23*** | 1.05±0.12*** | -1.26±0.88^ns^ | 0±0.85^ns^ | **22.54** | 0.96 |
| January 2008 | 2.4±0.43*** | 0.9±0.13*** | 42.81 | 0.85 | 3.07±0.37*** | 1.36±0.16*** | -1.39±0.42* | 0±0.83^ns^ | **35.2** | 0.95 |
| April 2008 | 3.7±0.46*** | 1.23±0.17*** | 34.46 | 0.89 | 8.51±2.1** | 3.58±0.82** | -2.74±0.83* | 0±0.41^ns^ | **21.53** | 0.98 |
| July 2008 | 3.56±0.73** | 1.15±0.26** | 27.77 | 0.8 | 3.23±0.34** | 2.05±0.28** | -2.33±0.53* | 0±0.43^ns^ | **17.16** | 0.97 |
| October 2008 | 3.17±0.51** | 1.12±0.18** | 22.7 | 0.89 | 2.97±0.42** | 1.52±0.24** | -1.43±1.1^ns^ | 0±1.23^ns^ | **19.84** | 0.96 |
|  |  |  |  |  |  |  |  |  |  |  |
| **Chordata** |  |  |  |  |  |  |  |  |  |  |
| January 2007 | 1.09±0.48^ns^ | 1.04±0.21** | **18.49** | 0.86 | - | - | - | - | - | - |
| May 2007 | 3.11±0.65* | 0.07±0.46^ns^ | 20.19 | 0.01 | 6.53±3.83^ns^ | 2.36±2.42^ns^ | -2.91±2.71^ns^ | 0±1.08^ns^ | **19.57** | 0.54 |
| August 2007 | 1.51±0.52* | 0.78±0.22* | **22.14** | 0.77 | - | - | - | - | - | - |
| November 2007 | 3.38±0.74** | 0.85±0.33^ns^ | 31.2 | 0.57 | 5.27±0.47** | 2.88±0.37** | -3.14±0.43** | 0±0.26^ns^ | **14.5** | 0.98 |
| January 2008 | 0.46±0.75^ns^ | 1.22±0.4^ns^ | **11.85** | 0.82 | - | - | - | - | - | - |
| April 2008 | 3.01±0.33*** | 0.3±0.16^ns^ | 16.82 | 0.47 | 2.35±1.72^ns^ | -0.35±1.09^ns^ | 0.93±1.12^ns^ | 0±1.95^ns^ | **16.28** | 0.73 |
| July 2008 | 1.51±0.6^ns^ | 1.04±0.2** | **21.83** | 0.87 | - | - | - | - | - | - |
| October 2008 | 2.02±0.55** | 0.88±0.17** | 34.68 | 0.8 | 4.06±2.37^ns^ | 2.81±1.5^ns^ | -2.23±1.51^ns^ | 0±0.89^ns^ | **32.26** | 0.9 |

**Table S5**: Relationship between log species richness and log abundance within individual based body size classes. We tested the fit of the linear model for the three dominant Phyla: Mollusca, Arthropoda and Chordata. Data for Equinodermata did not allow sufficient sample size for statistical testing and hence are not shown. The table shows the fitted values of the intercept (β_0_) and slope of the scaling region (β_1_). For the slope parameter, the 95% confidence intervals are shown in parentheses rather than standard errors (see text for details on theoretical expectations). Also shown are the Bayesian Information Criterion (BIC) and R^2^. Significance levels for the slope parameters are shown by the same abbreviations as in Table S2.

| Model | Linear: Y= β_0_+ β_1_X | | | |
| --- | --- | --- | --- | --- |
| Date | β_0_ | β_1_ | BIC | R^2^ |
| **Mollusca** |  |  |  |  |
| January 2007 | -0.09±0.18 ^ns^ | 0.08±0.06 ^ns^ | 10.51 | 0.24 |
| May 2007 | 0.41±0.35 ^ns^ | -0.02±0.08 ^ns^ | 9.63 | 0.01 |
| August 2007 | -0.04±0.32 ^ns^ | 0.06±0.07 ^ns^ | 10.69 | 0.15 |
| November 2007 | 0.02±0.24 ^ns^ | 0.11±0.05 ^ns^ | 11.19 | 0.48 |
| January 2008 | 0.28±0.31 ^ns^ | 0.02±0.06 ^ns^ | 19.52 | 0.01 |
| April 2008 | 0.01± ^ns^ | 0.1± ^ns^ | 0 | 0.38 |
| July 2008 | 0.29±0.45 ^ns^ | 0.04±0.07 ^ns^ | 10.95 | 0.08 |
| October 2008 | -0.05± ^ns^ | 0.09± ^ns^ | 0 | 0.33 |
|  |  |  |  |  |
| **Arthropoda** |  |  |  |  |
| January 2007 | 0.02±0.33 ^ns^ | 0.17±0.12 ^ns^ | 17.23 | 0.19 |
| May 2007 | 0.08±0.35 ^ns^ | 0.09±0.1 ^ns^ | 25.39 | 0.09 |
| August 2007 | -0.21±0.37 ^ns^ | 0.27±0.12 ^ns^ | 12.54 | 0.48 |
| November 2007 | 1.62±1.49 ^ns^ | -0.25±0.34 ^ns^ | 21.87 | 0.07 |
| January 2008 | 0.01±0.24 ^ns^ | 0.21±0.1 ^ns^ | 15.83 | 0.32 |
| April 2008 | -0.27± ^ns^ | 0.12± ^ns^ | 0 | 0.17 |
| July 2008 | 0.26±0.79 ^ns^ | 0.14±0.2 ^ns^ | 17.52 | 0.09 |
| October 2008 | -0.19 ^ns^ | 0.14 ^ns^ | 0 | 0.08 |
|  |  |  |  |  |
| **Chordata** |  |  |  |  |
| January 2007 | -0.04±0.21 ^ns^ | 0.27±0.17 ^ns^ | 6.1 | 0.38 |
| May 2007 | 0.35±0.41 ^ns^ | -0.02±0.12 ^ns^ | 8.15 | 0.01 |
| August 2007 | -0.04±0.14 ^ns^ | 0.13±0.09 ^ns^ | 3.47 | 0.36 |
| November 2007 | 0.77±0.59 ^ns^ | -0.04±0.17 ^ns^ | 18.67 | 0.01 |
| January 2008 | -0.07±0.29 ^ns^ | 0.69±0.31 ^ns^ | 4.52 | 0.71 |
| April 2008 | 0.02±0.01 ^ns^ | 0.14±0.00 ^ns^ | 0 | 0.16 |
| July 2008 | -0.11±0.32 ^ns^ | 0.41±0.19 ^ns^ | 9.92 | 0.53 |
| October 2008 | -0.21± ^ns^ | 0.47±*** | 0 | 0.86 |

**Table S6**: Relationship between log abundance and log species-averaged body size data. The table shows the estimated values for the 4-parameter Weibull function and the linear (power law) fit. Analysis are shown for the three dominant Phyla: Mollusca, Arthropoda and Chordata. In those cases where parameter estimates did not converge, no parameter values are shown and are indicated with a dash symbol (-). Data for Equinodermata did not allow sufficient sample size for statistical testing and hence are not shown. Also shown are the Bayesian Information Criterion (BIC) and R^2^ values. Minimum values of BIC are highlighted in bold, indicating the selected model. Significance levels for the parameters are shown by the same abbreviations as in Table S2. See text for details on the meaning of the different parameters.

| Model | Linear |  |  |  | Weibull |  |  |  |  |  |
| --- | --- | --- | --- | --- | --- | --- | --- | --- | --- | --- |
| Date | β_0_ | β_1_ | BIC | R^2^ | θ | α | γ | κ | BIC | R^2^ |
| **Mollusca** |  |  |  |  |  |  |  |  |  |  |
| January 2007 | 6.5 | -1.47 | - | 0.99 | - | - | - | - | - | - |
| May 2007 | 4.92±1.05* | -1.07±0.41* | **17** | 0.77 | -0.85 | 9.34 | 1.72 | 1.08 | - | 0 |
| August 2007 | 5.63±0.94^ns^ | -1.09±0.32* | **10.74** | 0.92 | 0 | 6.73 | 0.95 | 1.54 | - | 0 |
| November 2007 | 6.21±0.15* | -1.27±0.06* | **-0.25** | 1.00 | -1.18 | 7.36 | 3.65 | 1.65 | - | 0 |
| January 2008 | 6.5±0.1* | -1.47 | **22.38** | 1.00 | -0.5 | 18.68 | 1.47 | 4.01 | - | 0 |
| April 2008 | 4.92±1.05* | -1.07±0.41^ns^ | **14.69** | 0.77 | -0.9 | 13.17 | 1.04 | 1.08 | - | 0 |
| July 2008 | 5.63±0.94^ns^ | -1.09±0.32^ns^ | **3.89** | 0.92 | - | - | - | - | - | - |
| October 2008 | 6.21±0.15* | -1.27±0.06* | **18.12** | 1.00 | -0.93 | 11.89 | 1.62 | 1.04 | - | 0 |
|  |  |  |  |  |  |  |  |  |  |  |
| **Arthropoda** |  |  |  |  |  |  |  |  |  |  |
| January 2007 | 1.63±0.59* | 0.06±0.21^ns^ | **38.92** | 0.01 | -0.49±0.57^ns^ | 5.82±0.72*** | 3.24±0.71** | 1.67±0.59* | 8.22 | 0.79 |
| May 2007 | 2.79±0.89* | -0.05±0.32^ns^ | 46.37 | 0.00 | 0.28±0.42^ns^ | 6.18±0.56** | 33.74±3E02^ns^ | 19.1±2E02^ns^ | **3.57** | 0.9 |
| August 2007 | 4.23±1.25* | -0.6±0.44^ns^ | **35.25** | 0.27 | -0.13±0.54^ns^ | 6.74±1.29* | 2E05±7E08^ns^ | 9E04±5E07^ns^ | 11.1 | 0.7 |
| November 2007 | 2.75±1.09^ns^ | -0.08±0.38^ns^ | 33.3 | 0.01 | -0.54±0.35ns | 7.23±0.84** | 2E05±8E07^ns^ | 1E05±5E07^ns^ | **8.82** | 0.88 |
| January 2008 | 1.63±0.59* | 0.06±0.21^ns^ | **35.73** | 0.01 | -0.68±0.19* | 8.04±0.35*** | 12.4±5.82^ns^ | 5.69±2.69^ns^ | -1.33 | 0.96 |
| April 2008 | 2.79±0.89* | -0.05±0.32^ns^ | **35.72** | 0.00 | -0.28±0.74ns | 6.7±0.81* | 6E04±3E08^ns^ | 3E04±2E08^ns^ | 0.17 | 0.97 |
| July 2008 | 4.23±1.25* | -0.6±0.44^ns^ | **34.86** | 0.27 | -1.56±0.08** | 8.66±0.31** | 3.32±0.26** | 1.26±0.15* | -8.4 | 0.99 |
| October 2008 | 2.75±1.09^ns^ | -0.08±0.38^ns^ | **12.24** | 0.01 | -0.78±0.18* | 8.21±0.38** | 4.32±1.7^ns^ | 2.15±0.77^ns^ | -1.73 | 0.98 |
|  |  |  |  |  |  |  |  |  |  |  |
| **Chordata** |  |  |  |  |  |  |  |  |  |  |
| January 2007 | 1.09±0.17* | -0.06±0.29^ns^ | **-** | 0.02 | - | - | - | - | - | - |
| May 2007 | 3.88±0.13* | -1.64±0.09* | **-1.41** | 1.00 | - | - | - | - | - | - |
| August 2007 | 3.11±0.12* | -0.33 | **-** | 1.00 | - | - | - | - | - | - |
| November 2007 | 3.9±1.35^ns^ | -0.78±0.54^ns^ | **23.78** | 0.41 | - | - | - | - | - | - |
| January 2008 | 1.58±1.99^ns^ | -0.35±0.92^ns^ | **10.11** | 0.12 | - | - | - | - | - | - |
| April 2008 | 3.71±0.53** | -0.99±0.23^ns^ | **7.12** | 0.98 | - | - | - | - | - | - |
| July 2008 | 1.87±1.21^ns^ | -0.06±0.44^ns^ | **56.45** | 0.01 | - | - | - | - | - | - |
| October 2008 | 2.95±1.21^ns^ | -0.12±0.44^ns^ | **76.12** | 0.01 | - | - | - | - | - | - |

**Table S7**: Relationship between log species richness and log species-averaged body size data. The table shows the estimated values for the 4-parameter Weibull function and the linear (power law) fit. Analysis are shown for three dominant Phyla: Mollusca, Arthropoda and Chordata. In those cases where parameter estimates did not converge, no parameter values are shown and are indicated with a dash symbol (-). Data for Equinodermata did not allow sufficient sample size for statistical testing and hence are not shown. Also shown are the Bayesian Information Criterion (BIC) and R^2^ values. Minimum values of BIC are highlighted in bold, indicating the selected model. Significance levels for the parameters are shown by the same abbreviations as in Table S2. See text for details on the meaning of the different parameters.

| Model | Linear |  |  |  | Weibull |  |  |  |  |  |
| --- | --- | --- | --- | --- | --- | --- | --- | --- | --- | --- |
| Date | β_0_ | β_1_ | BIC | R^2^ | θ | α | γ | κ | BIC | R^2^ |
| **Mollusca** |  |  |  |  |  |  |  |  |  |  |
| January 2007 | 1.1±^ns^ | -0.37±^ns^ | **0** | 1 | - | - | - | - | - | - |
| May 2007 | 0.61±0.24^ns^ | -0.06±0.09^ns^ | **5.11** | 0.18 | - | - | - | - | - | - |
| August 2007 | 0.46±0.54^ns^ | -0.07±0.18^ns^ | **7.45** | 0.13 | - | - | - | - | - | - |
| November 2007 | 1.05±0.04* | -0.09±0.02^ns^ | **-8.12** | 0.96 | - | - | - | - | - | - |
| January 2008 | 1.1^ns^ | -0.37^ns^ | **8.21** | 1 | - | - | - | - | - | - |
| April 2008 | 0.61±0.24^ns^ | -0.06±0.09^ns^ | **-0.62** | 0.18 | - | - | - | - | - | - |
| July 2008 | 0.46±0.54^ns^ | -0.07±0.18^ns^ | **-0.09** | 0.13 | - | - | - | - | - | - |
| October 2008 | 1.05±0.04* | -0.09±0.02^ns^ | **-0.62** | 0.96 | - | - | - | - | - | - |
|  |  |  |  |  |  |  |  |  |  |  |
| **Arthopoda** |  |  |  |  |  |  |  |  |  |  |
| January 2007 | 0.79±0.92^ns^ | -0.03±0.3^ns^ | **13.33** | 0 | - | - | - | - | - | - |
| May 2007 | 0.29±0.38^ns^ | 0.11±0.17^ns^ | **18.77** | 0.09 | - | - | - | - | - | - |
| August 2007 | 0.33±0.56^ns^ | 0.1±0.18^ns^ | **12.95** | 0.12 | - | - | - | - | - | - |
| November 2007 | 0.29±0.52^ns^ | 0.05±0.15^ns^ | **16.3** | 0.04 | - | - | - | - | - | - |
| January 2008 | 0.79^ns^ | -0.03±^ns^ | **14.15** | 0 | - | - | - | - | - | - |
| April 2008 | 0.29±0.38^ns^ | 0.11±0.17^ns^ | **0** | 0.09 | - | - | - | - | - | - |
| July 2008 | 0.33±0.56^ns^ | 0.1±0.18^ns^ | **13.6** | 0.12 | - | - | - | - | - | - |
| October 2008 | 0.29±0.52^ns^ | 0.05±0.15^ns^ | **0** | 0.04 | - | - | - | - | - | - |
|  |  |  |  |  |  |  |  |  |  |  |
| **Chordata** |  |  |  |  |  |  |  |  |  |  |
| January 2007 | 0.42±0.23^ns^ | -0.09±0.09^ns^ | **6.88** | 0.23 | - | - | - | - | - | - |
| May 2007 | 0.59±0.24^ns^ | -0.2±0.17^ns^ | **2.56** | 0.57 | - | - | - | - | - | - |
| August 2007 | 0.69^ns^ | 0^ns^ | **0** | 0 | - | - | - | - | - | - |
| November 2007 | 0.83±0.56^ns^ | -0.06±0.23^ns^ | **15.06** | 0.03 | - | - | - | - | - | - |
| January 2008 | 0.42±0.23^ns^ | -0.09±0.09^ns^ | **7.86** | 0.23 | - | - | - | - | - | - |
| April 2008 | 0.59±0.24^ns^ | -0.2±0.17^ns^ | - | 0.57 | - | - | - | - | - | - |
| July 2008 | 0.69^ns^ | 0 ^ns^ | **9.37** | 0 | - | - | - | - | - | - |
| October 2008 | 0.83±0.56^ns^ | -0.06±0.23^ns^ | - | 0.03 | - | - | - | - | - | - |

**Table S8**: Relationship between log energy use and species-averaged log body size. We tested the fit of the linear and piecewise scaling models for the three dominant Phyla: Mollusca, Arthropoda and Chordata. In those cases where parameter estimates did not converge, no parameter values are shown and are indicated with a dash symbol (-). Data for Equinodermata did not allow sufficient sample size for statistical testing and hence are not shown. The table shows the fitted values of the intercept (β_0_), slope of the first scaling region (β_1_), slope of the second scaling region (β_2_) and the threshold value (τ). Also shown are the Bayesian Information Criterion (BIC) and R^2^. Minimum values of BIC are highlighted in bold, indicating the selected model. Significance levels for the slope parameters are shown by the same abbreviations as in Table S2.

| Model | Linear: Y= β_0_ + β_1_X | |  |  | Piecewise: Y= β_0_ + β_1_X - β_2_(X-τ)+ | |  |  |  |  |
| --- | --- | --- | --- | --- | --- | --- | --- | --- | --- | --- |
| Date | β_0_ | β_1_ | BIC | R^2^ | β_0_ | β_1_ | β_2_ | τ | BIC | R^2^ |
| **Mollusca** |  |  |  |  |  |  |  |  |  |  |
| January 2007 | 6.51±^ns^ | -0.29^ns^ | - | 1 | - | - | - | - | - | - |
| May 2007 | 5.1±1.24^ns^ | -0.15±0.49^ns^ | **18.31** | 0.05 | - | - | - | - | - | - |
| August 2007 | 5.85±1.24^ns^ | -0.08±0.42^ns^ | **12.43** | 0.04 | - | - | - | - | - | - |
| November 2007 | 6.47±0.37* | -0.3±0.15^ns^ | **5.19** | 0.79 | - | - | - | - | - | - |
| January 2008 | 6.09±2^ns^ | -0.79±1.2^ns^ | **22.9** | 0.18 | - | - | - | - | - | - |
| April 2008 | 6.08±0.8* | -0.47±0.31^ns^ | **14.8** | 0.53 | - | - | - | - | - | - |
| July 2008 | 7.33±0.03** | -0.3±0.02* | **-9.77** | 1 | - | - | - | - | - | - |
| October 2008 | 7.14±1.04* | -0.68±0.41^ns^ | **16.87** | 0.59 | - | - | - | - | - | - |
|  |  |  |  |  |  |  |  |  |  |  |
| **Arthropoda** |  |  |  |  |  |  |  |  |  |  |
| January 2007 | 3.66±1.39^ns^ | 0.7±0.45^ns^ | **16.65** | 0.55 | - | - | - | - | - | - |
| May 2007 | 2.12±1.03^ns^ | 1.03±0.45^ns^ | **30.82** | 0.56 | - | - | - | - | - | - |
| August 2007 | 2.07±1.74^ns^ | 1.09±0.56^ns^ | **22.03** | 0.66 | - | - | - | - | - | - |
| November 2007 | 2.92±1.72^ns^ | 0.88±0.5^ns^ | 28.32 | 0.51 | 5.16±2.41^ns^ | 2.34±1.13^ns^ | -4.36±2.04^ns^ | 0±1.3^ns^ | **22.97** | 0.91 |
| January 2008 | 3.47±0.68* | 0.8±0.23* | **19.41** | 0.8 | - | - | - | - | - | - |
| April 2008 | 2.82^ns^ | 2.26^ns^ | - | 1 | - | - | - | - | - | - |
| July 2008 | 2.49±2.15^ns^ | 1.3±0.7^ns^ | **20.1** | 0.63 | - | - | - | - | - | - |
| October 2008 | 1.98^ns^ | 2.33^ns^ | - | 1 | - | - | - | - | - | - |
| **Chordata** |  |  |  |  |  |  |  |  |  |  |
| January 2007 | 1.18±0.78^ns^ | 1.01±0.32^ns^ | **19.3** | 0.77 | 1.21±1.03^ns^ | 1.56±0.8^ns^ | -1.62±2.57^ns^ | 0±2.49^ns^ | 19.64 | 0.87 |
| May 2007 | 4.06±0.36^ns^ | -0.88±0.25^ns^ | **4.87** | 0.92 | - | - | - | - | - | - |
| August 2007 | 3.51±^ns^ | 0.48^ns^ | - | 1 | - | - | - | - | - | - |
| November 2007 | 4.18±1.33^ns^ | 0.1±0.54^ns^ | **23.68** | 0.01 | - | - | - | - | - | - |
| January 2008 | 1.53±2.41^ns^ | 0.64±1.11^ns^ | **11.24** | 0.25 | - | - | - | - | - | - |
| April 2008 | 4.1±0.54^ns^ | 0.02±0.22^ns^ | **7.02** | 0.01 | - | - | - | - | - | - |
| July 2008 | 2.44±1.29^ns^ | 0.85±0.47^ns^ | **17.26** | 0.62 | - | - | - | - | - | - |
| October 2008 | 3.27±1.35^ns^ | 0.79±0.49^ns^ | **17.66** | 0.56 | - | - | - | - | - | - |

**Table S9**: Relationship between log species richness and log abundance within species - averaged body size classes. We tested the fit of the linear model for the three dominant Phyla: Mollusca, Arthropoda and Chordata. Data for Equinodermata did not allow sufficient sample size for statistical testing and hence are not shown. The table shows the fitted values of the intercept (β_0_) and slope of the scaling region (β_1_), together with associated standard errors (see text for details on theoretical expectations). Also shown are the Bayesian Information Criterion (BIC) and R^2^. Significance levels for the slope parameters are shown by the same abbreviations as in Table S2.

| Model | Linear: Y= β_0_+ β_1_X | | | |
| --- | --- | --- | --- | --- |
| Date | β_0_ | β_1_ | BIC | R^2^ |
| **Mollusca** |  |  |  |  |
| January 2007 | -0.52^ns^ | 0.25^ns^ | - | 1 |
| May 2007 | 0.28±0.28^ns^ | 0.07±0.07^ns^ | 4.08 | 0.36 |
| August 2007 | -0.07±0.71^ns^ | 0.11±0.14^ns^ | 6.48 | 0.37 |
| November 2007 | 0.62±0.06^ns^ | 0.07±0.01^ns^ | -9.88 | 0.98 |
| January 2008 | 0.22±0.2^ns^ | 0.11±0.03^ns^ | 1.84 | 0.84 |
| April 2008 | -0.05^ns^ | 0.17±** | 0 | 0.99 |
| July 2008 | 0.47±0.33^ns^ | 0.06±0.05^ns^ | -0.77 | 0.59 |
| October 2008 | -0.13^ns^ | 0.15±* | 0 | 0.94 |
|  |  |  |  |  |
| **Arthropoda** |  |  |  |  |
| January 2007 | -0.5^ns^ | 0.44^ns^ | 8.39 | 0.71 |
| May 2007 | -0.38±0.14^ns^ | 0.36±0.05** | 3.22 | 0.93 |
| August 2007 | -0.13±0.49^ns^ | 0.26±0.15^ns^ | 9.9 | 0.59 |
| November 2007 | -0.1±0.43^ns^ | 0.19±0.11^ns^ | 13.13 | 0.49 |
| January 2008 | -0.53±0.58^ns^ | 0.4±0.18^ns^ | 9.4 | 0.63 |
| April 2008 | -1.63^ns^ | 0.43^ns^ | 0 | 1 |
| July 2008 | -0.32±0.72^ns^ | 0.31±0.2^ns^ | 10.44 | 0.55 |
| October 2008 | -0.36^ns^ | 0.26^ns^ | 0 | 1 |
| **Chordata** |  |  |  |  |
| January 2007 | -0.02^ns^ | 0.3^ns^ | 2.56 | 0.68 |
| May 2007 | 0.14±0.38^ns^ | 0.11±0.11^ns^ | 2.91 | 0.52 |
| August 2007 | 0.69^ns^ | 0^ns^ | - | 0 |
| November 2007 | 0.11±0.41^ns^ | 0.24±0.12^ns^ | 11.07 | 0.56 |
| January 2008 | -0.16±0.27^ns^ | 0.6±0.23^ns^ | 1.59 | 0.88 |
| April 2008 | 0.05^ns^ | 0.27^ns^ | 0 | 0.99 |
| July 2008 | -0.18±0.31^ns^ | 0.42±0.15^ns^ | 4.17 | 0.81 |
| October 2008 | -0.42^ns^ | 0.5^ns^ | 0 | 0.88 |

**Appendix S2:**

**Supporting Figures**

| 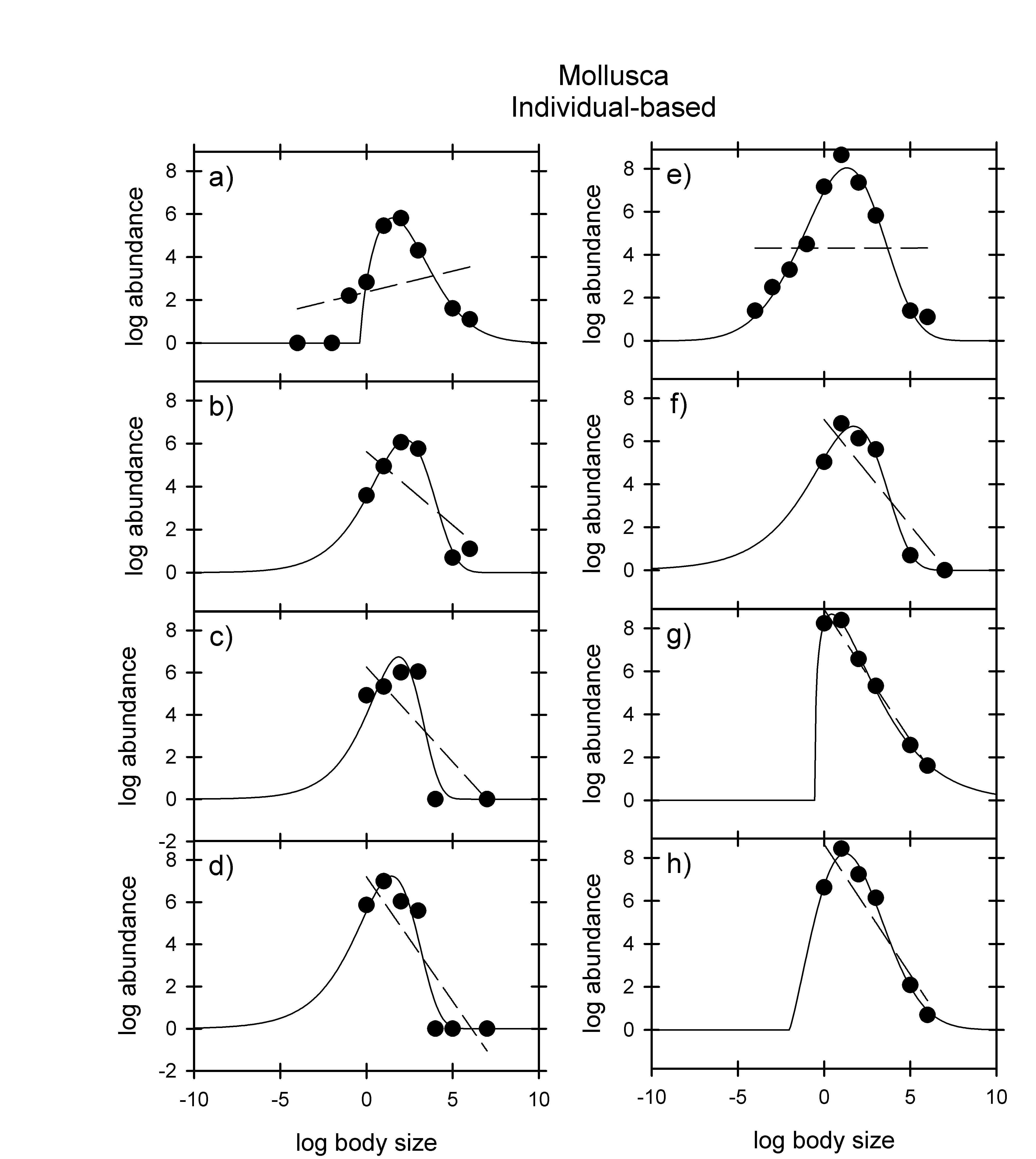 |
| --- |
| **Figure S1:** Temporal dynamics in the relationship between log abundance and log individual-based body size classes in Phylum Mollusca. Figures a to d show the observed values and fitted power law (dashed lines) log-Weibull functions (continuous curves) for log abundance in January, May, August and November 2007 respectively, while figures e to h show the observed values and corresponding fitted functions for January, April, July and October 2008, respectively. Parameter values and fitted R^2^ values are shown in Supporting Table S2, Appendix S1. |

| 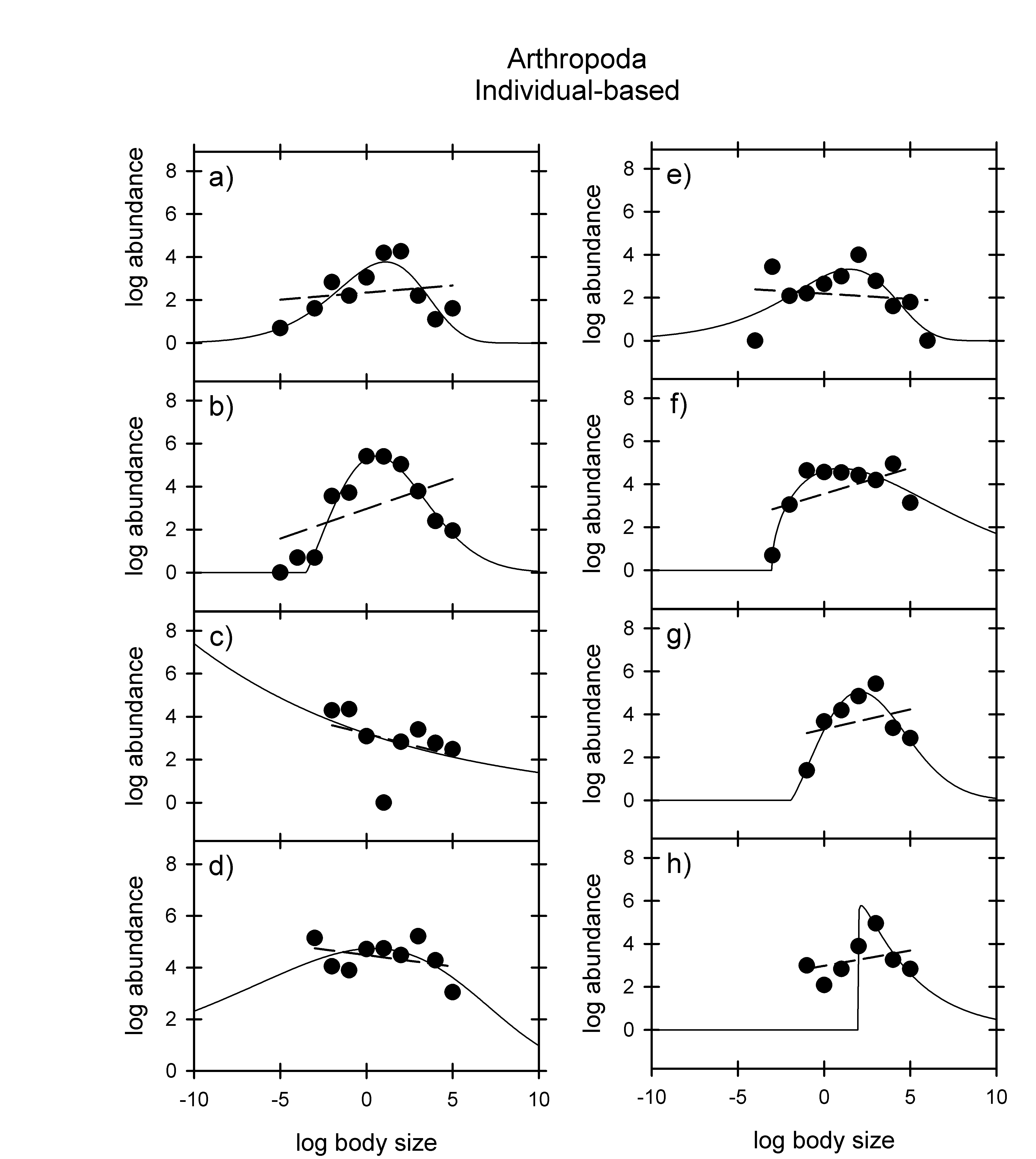 |
| --- |
| **Figure S2:** Temporal dynamics in the relationship between log abundance and log individual-based body size classes in Phylum Arthropoda. Figures a to d show the observed values and fitted power law (dashed lines) log-Weibull functions (continuous curves) for log abundance in January, May, August and November 2007 respectively, while figures e to h show the observed values and corresponding fitted functions for January, April, July and October 2008, respectively. Parameter values and fitted R^2^ values are shown in Supporting Table S2, Appendix S1. |

| 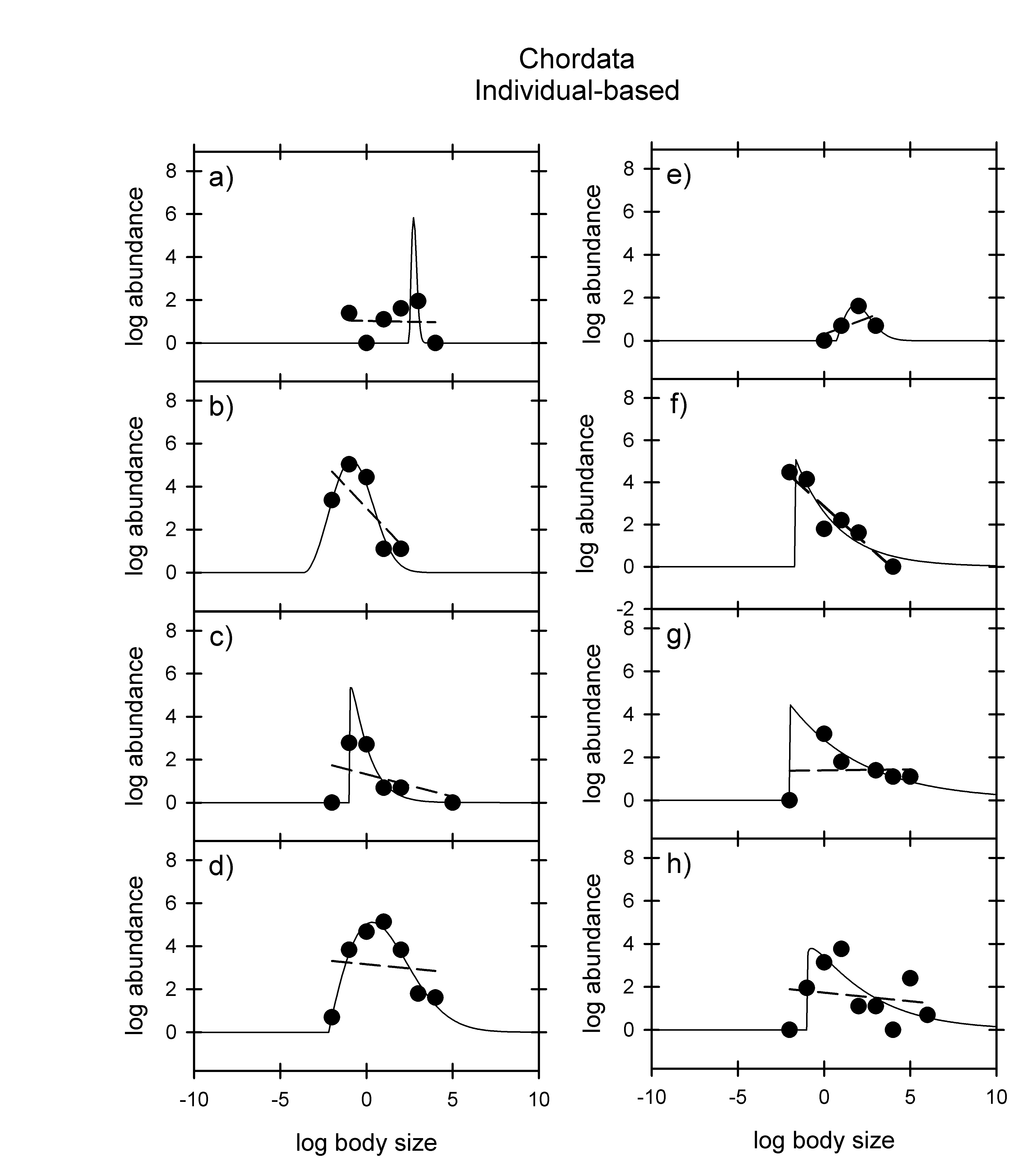 |
| --- |
| **Figure S3:** Temporal dynamics in the relationship between log abundance and log individual-based body size classes in Phylum Chordata. Figures a to d show the observed values and fitted power law (dashed lines) log-Weibull functions (continuous curves) for log abundance in January, May, August and November 2007 respectively, while figures e to h show the observed values and corresponding fitted functions for January, April, July and October 2008, respectively. Parameter values and fitted R^2^ values are shown in Supporting Table S2, Appendix S1. |

| 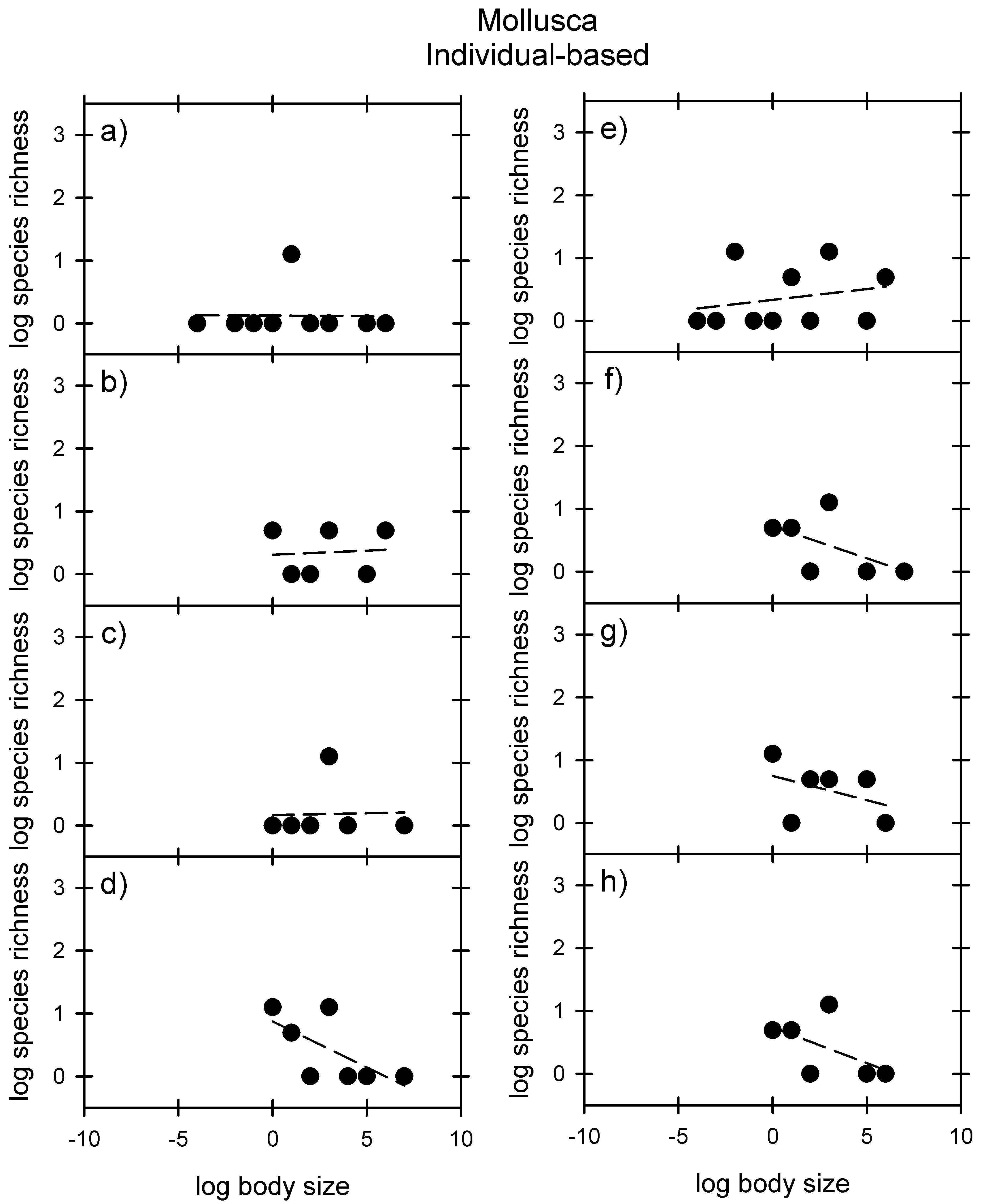 |
| --- |
| **Figure S4:** Temporal dynamics in the relationship between log species richness and individual based body size classes in Phylum Mollusca. Figures a to d show the observed values and fitted power law (dashed lines) log-Weibull functions (continuous curves) for log abundance in January, May, August and November 2007 respectively, while figures e to h show the observed values and corresponding fitted functions for January, April, July and October 2008, respectively. Parameter values and fitted R^2^ values are shown in Supporting Table S3, Appendix S1. |

| 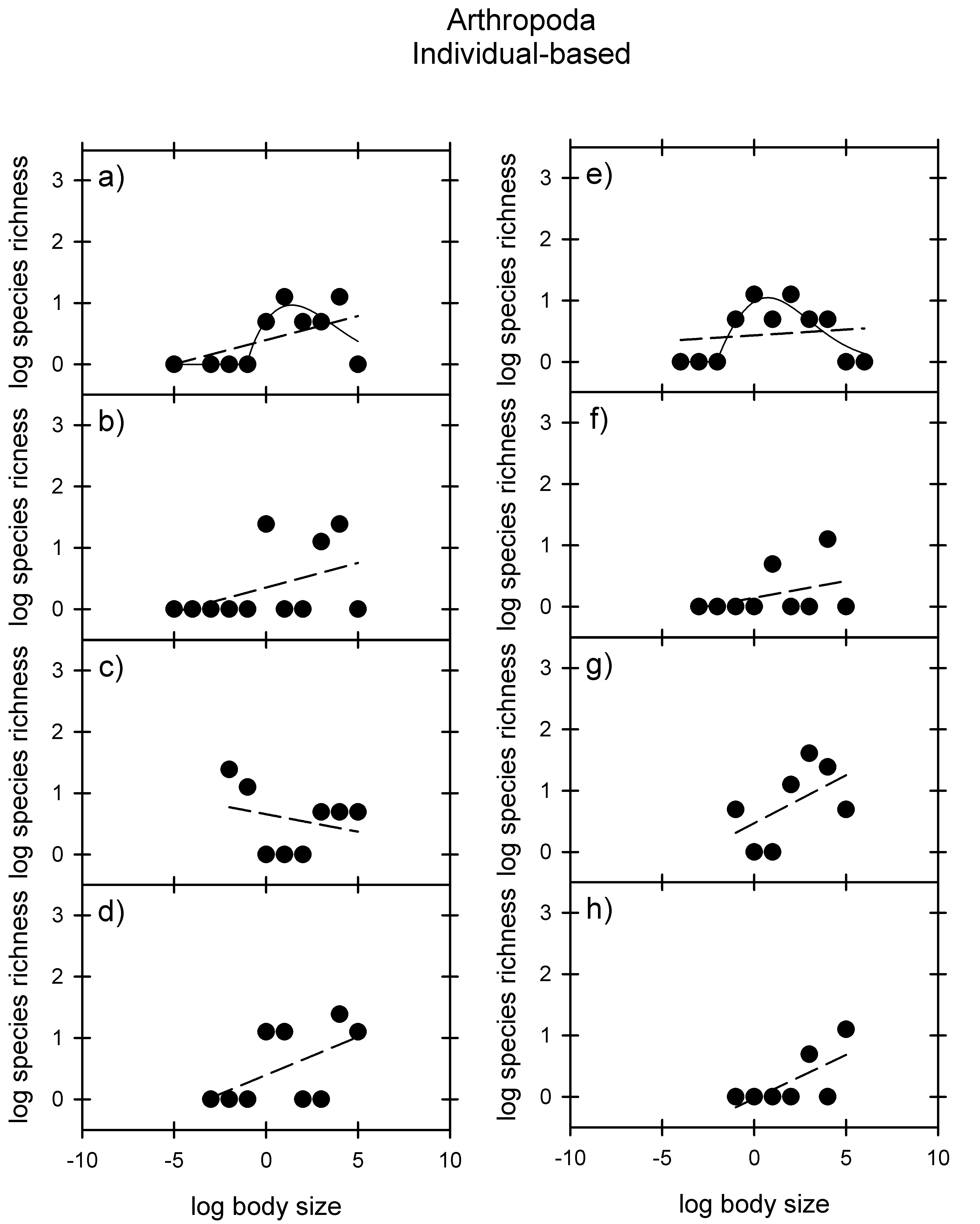 |
| --- |
| **Figure S5:** Temporal dynamics in the relationship between log species richness and log individual based body size classes in Phylum Arthropoda. Figures a to d show the observed values and fitted power law (dashed lines) log-Weibull functions (continuous curves) for log abundance in January, May, August and November 2007 respectively, while figures e to h show the observed values and corresponding fitted functions for January, April, July and October 2008, respectively. Parameter values and fitted R^2^ values are shown in Supporting Table S3, Appendix S1. |

| 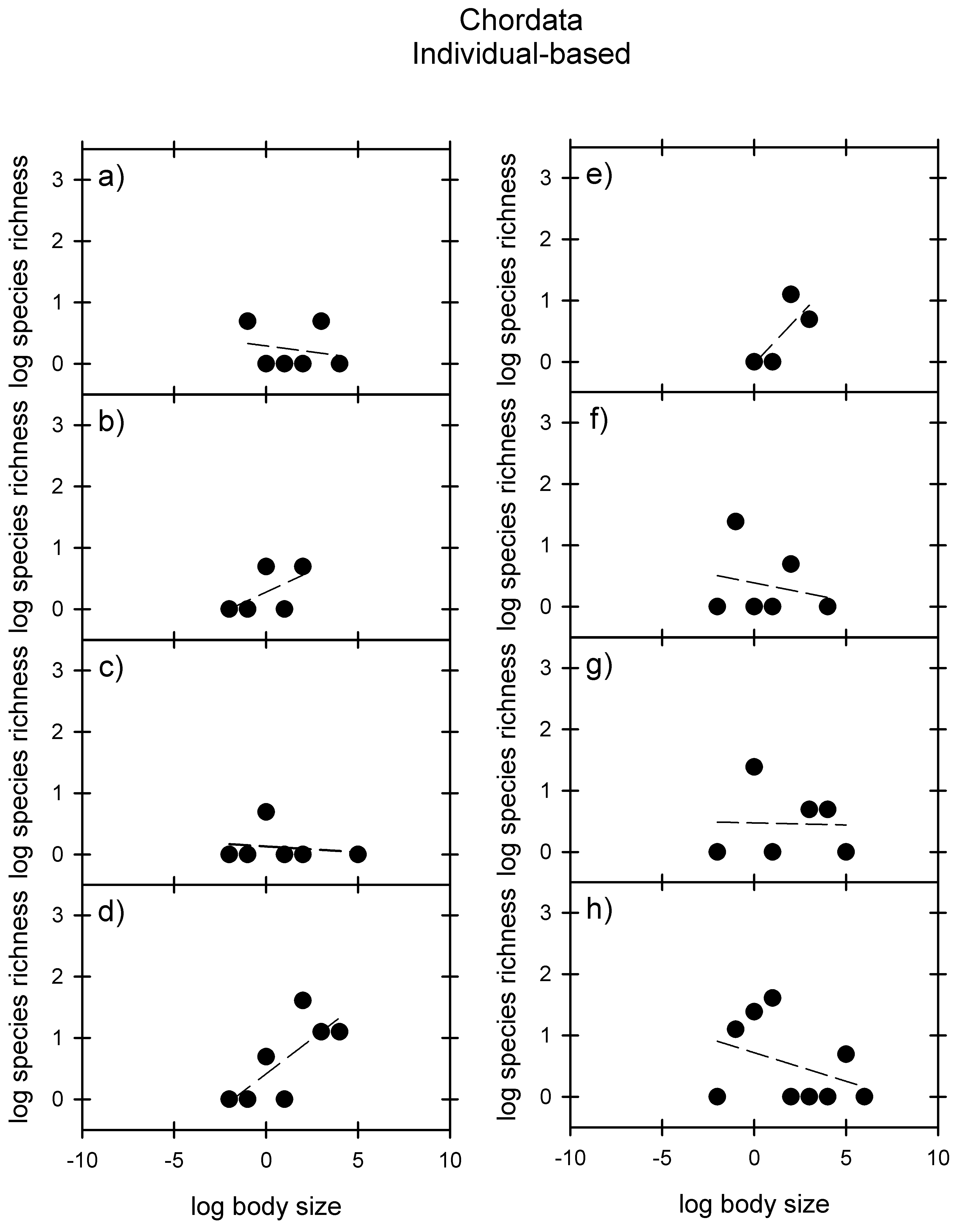 |
| --- |
| **Figure S6:** Temporal dynamics in the relationship between log species richness and log individual based body size classes in Phylum Chordata. Figures a to d show the observed values and fitted power law (dashed lines) log-Weibull functions (continuous curves) for log abundance in January, May, August and November 2007 respectively, while figures e to h show the observed values and corresponding fitted functions for January, April, July and October 2008 respectively. Parameter values and fitted R^2^ values are shown in Supporting Table S3, Appendix S1. |

| 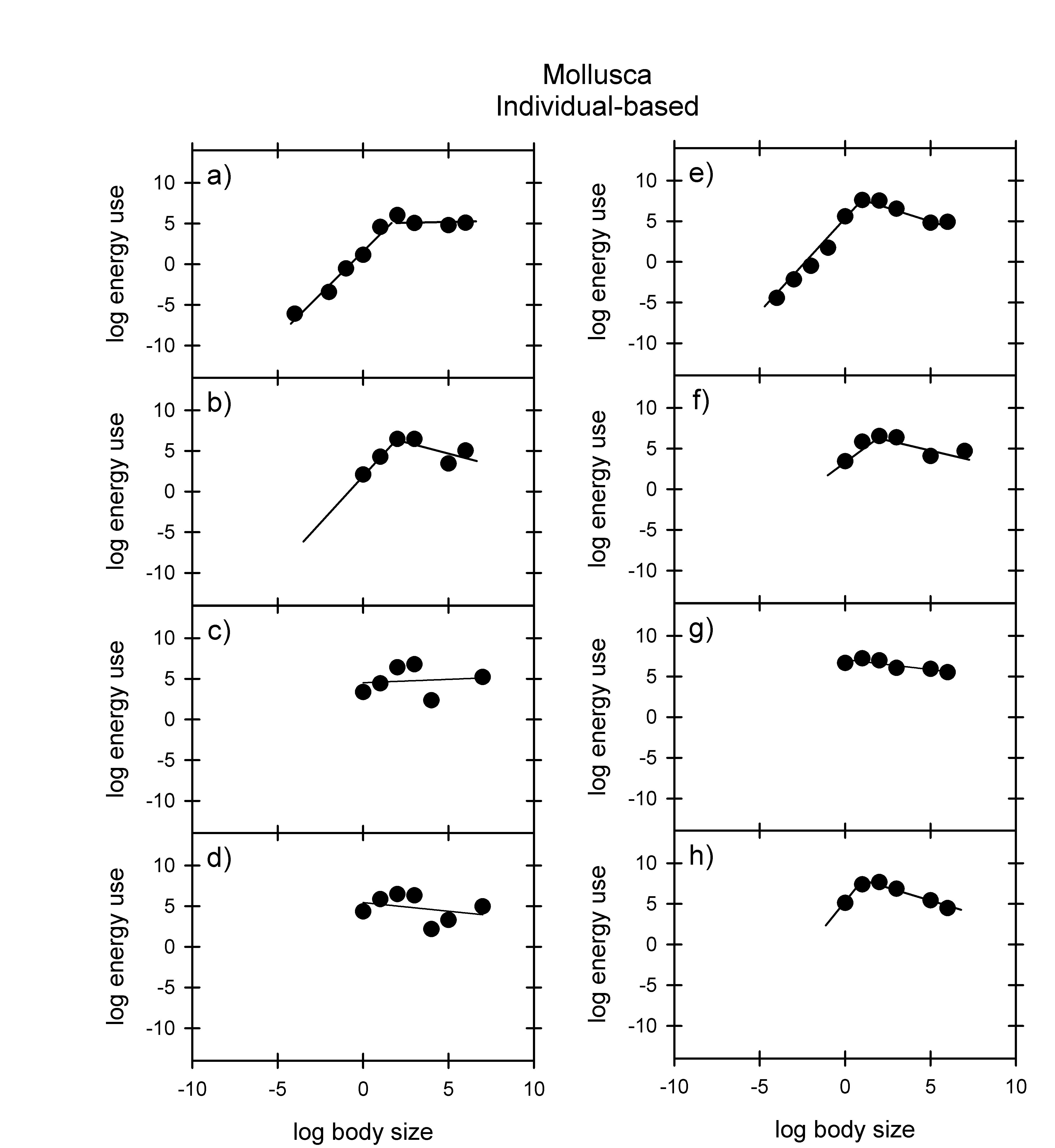 |
| --- |
| **Figure S7:** The figure shows temporal dynamics of the observed scaling of log total energy use (Watts) as a function of log individual based body size classes in Phylum Mollusca. Filled circles show the total energy use in each body size class. Continuous lines show the best fitted regressions. Figures a to d show the observed values and fitted functions for log energy use in January, May, August and November 2007 respectively, while figures e to h show the observed values and fitted functions for January, April, July and October 2008, respectively. Parameter values and fitted R^2^ values are shown in Supporting Table S4, Appendix S1. |

| 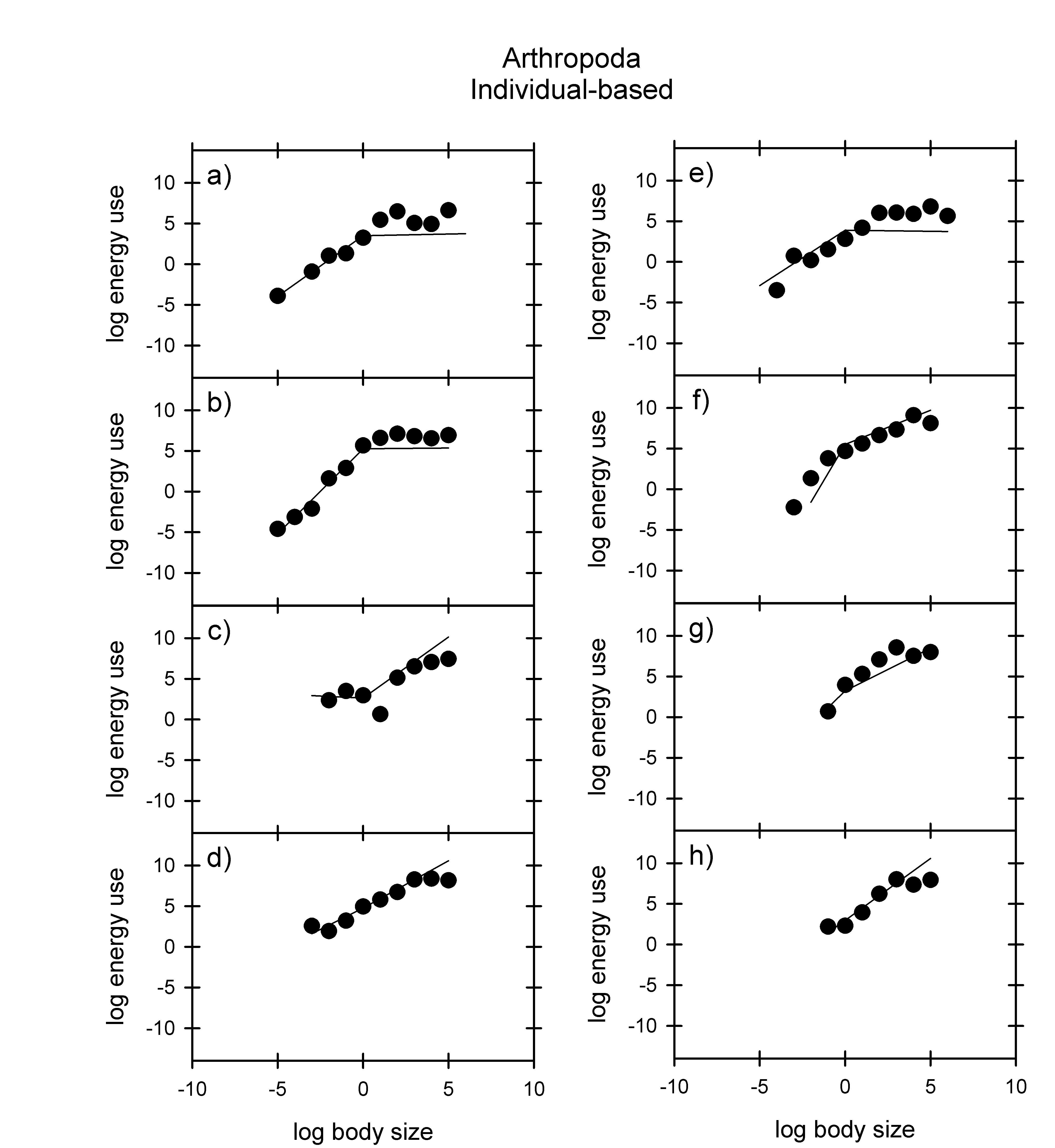 |
| --- |
| **Figure S8:** The figure shows temporal dynamics of the observed scaling of log total energy use (Watts) as a function of log individual based body size classes in Phylum Arthropoda. Filled circles show the total energy use in each body size class. Continuous lines show the best fitted regressions. Figures a to d show the observed values and fitted functions for log energy use in January, May, August and November 2007 respectively, while figures e to h show the observed values and fitted functions for January, April, July and October 2008, respectively. Parameter values and fitted R^2^ values are shown in Supporting Table S4, Appendix S1. |

| 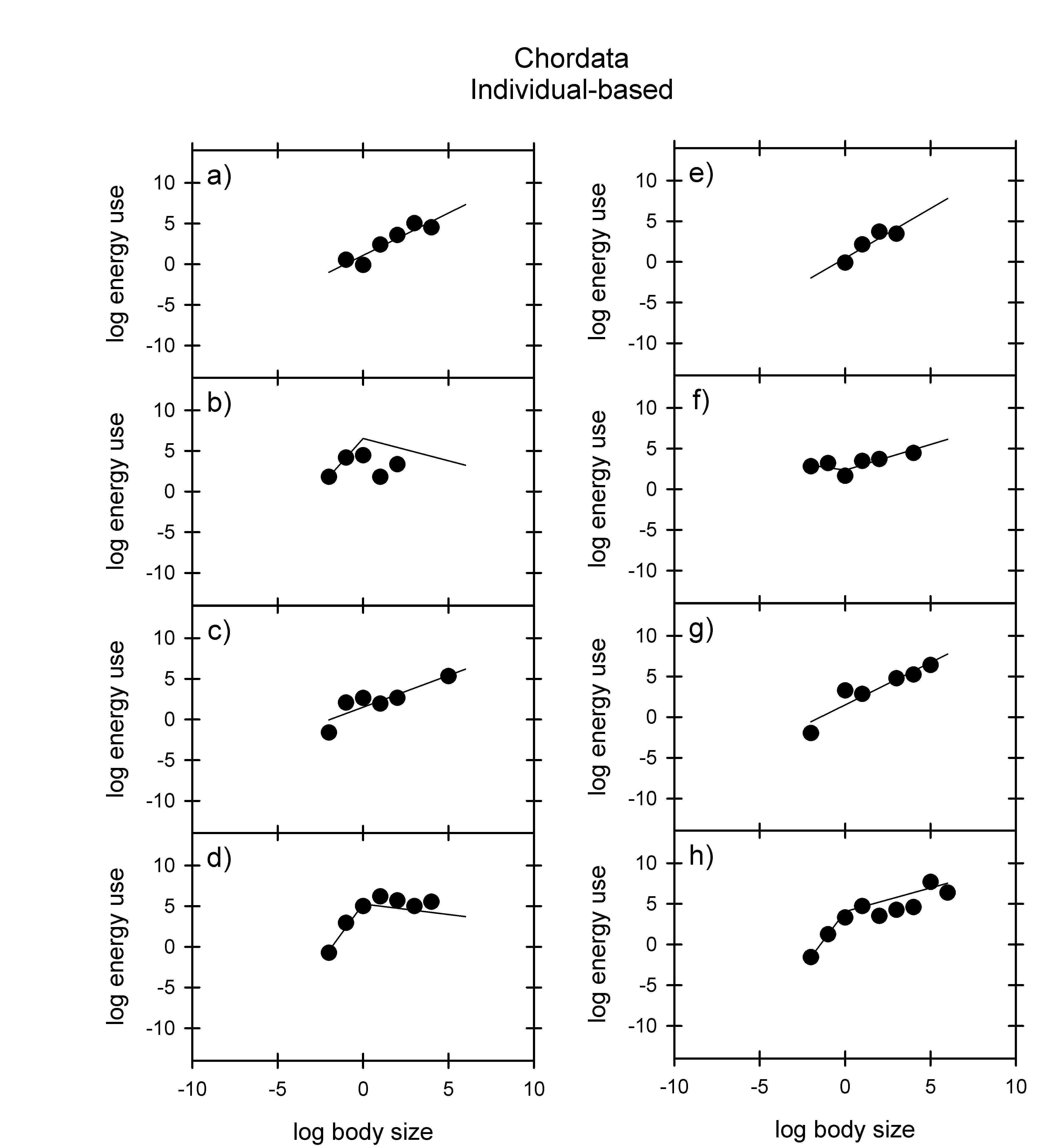 |
| --- |
| **Figure S9:** The figure shows temporal dynamics of the observed scaling of log total energy use (Watts) as a function of log individual based body size classes in Phylum Chordata. Filled circles show the total energy use in each body size class. Continuous lines show the best fitted regressions. Figures a to d show the observed values and fitted functions for log energy use in January, May, August and November 2007 respectively, while figures e to h show the observed values and fitted functions for January, April, July and October 2008, respectively. Parameter values and fitted R^2^ values are shown in Supporting Table S4, Appendix S1. |

| 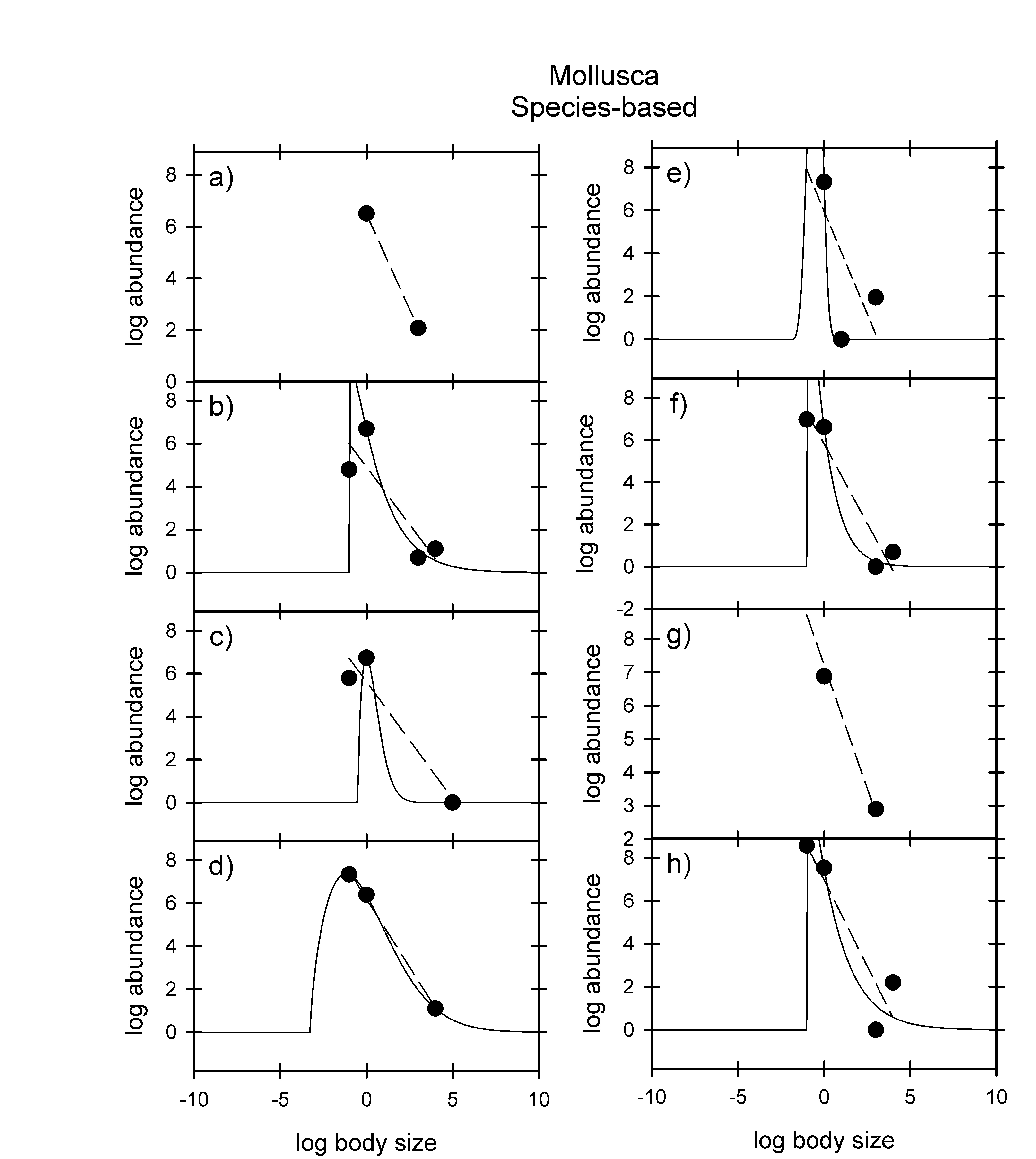 |
| --- |
| **Figure S10:** Temporal dynamics in the relationship between log abundance and species averaged log body size classes in Phylum Mollusca. Figures a to d show the observed values and fitted power law (dashed lines) log-Weibull functions (continuous curves) for log abundance in January, May, August and November 2007 respectively, while figures e to h show the observed values and corresponding fitted functions for January, April, July and October 2008, respectively. Parameter values and fitted R^2^ values are shown in Supporting Table S6, Appendix S1. |

| 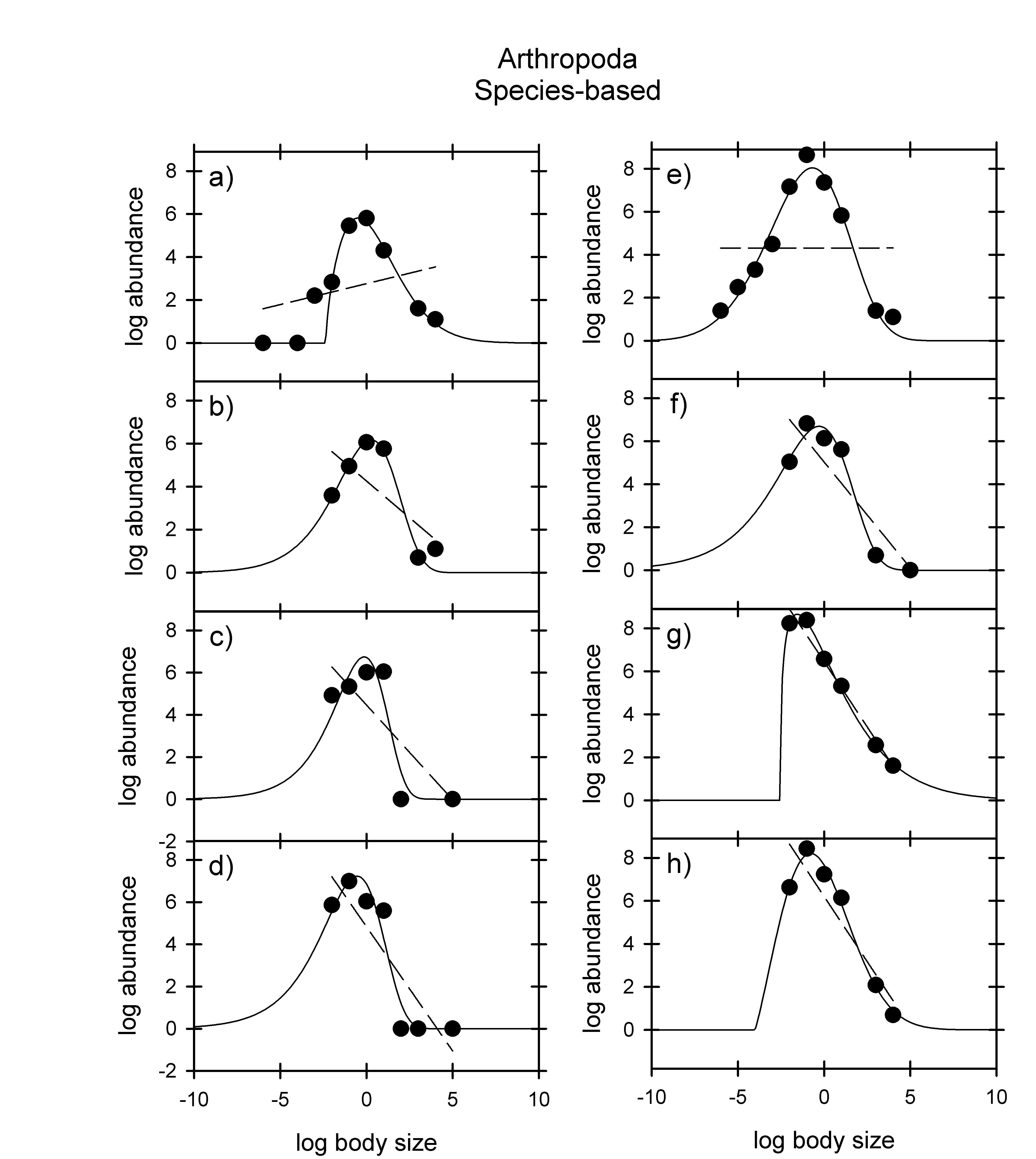 |
| --- |
| **Figure S11:** Temporal dynamics in the relationship between log abundance and species averaged log body size classes in Phylum Arthropoda. Figures a to d show the observed values and fitted power law (dashed lines) log-Weibull functions (continuous curves) for log abundance in January, May, August and November 2007 respectively, while figures e to h show the observed values and corresponding fitted functions for January, April, July and October 2008, respectively. Parameter values and fitted R^2^ values are shown in Supporting Table S6, Appendix S1. |

| 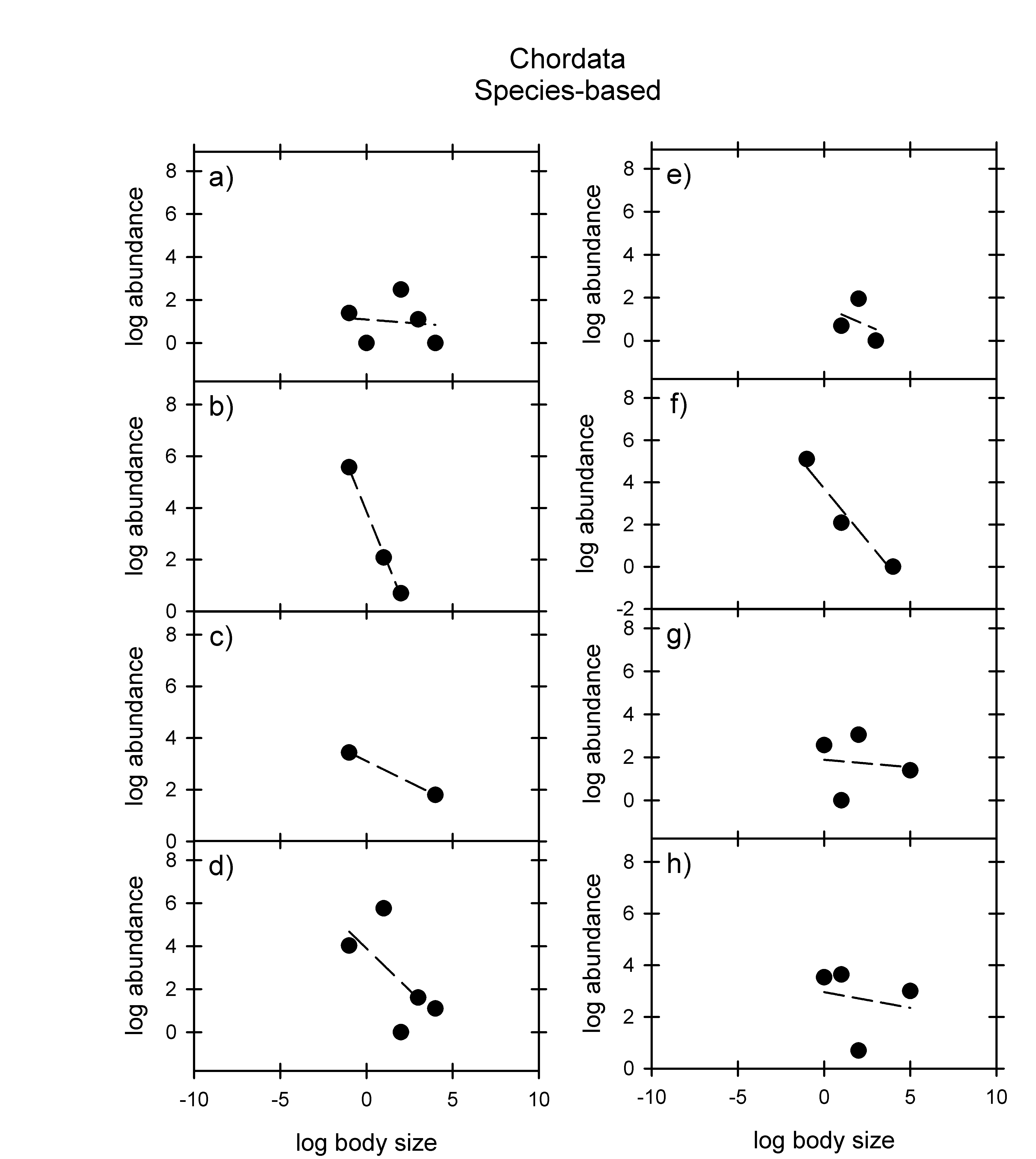 |
| --- |
| **Figure S12:** Temporal dynamics in the relationship between log abundance and species averaged log body size classes in Phylum Chordata. Figures a to d show the observed values and fitted power law (dashed lines) for log abundance in January, May, August and November 2007 respectively, while figures e to h show the observed values and corresponding fitted functions for January, April, July and October 2008, respectively. Log-Weibull functions could not be fitted and hence are not shown. Parameter values and fitted R^2^ values are shown in Supporting Table S6, Appendix S1. |

| 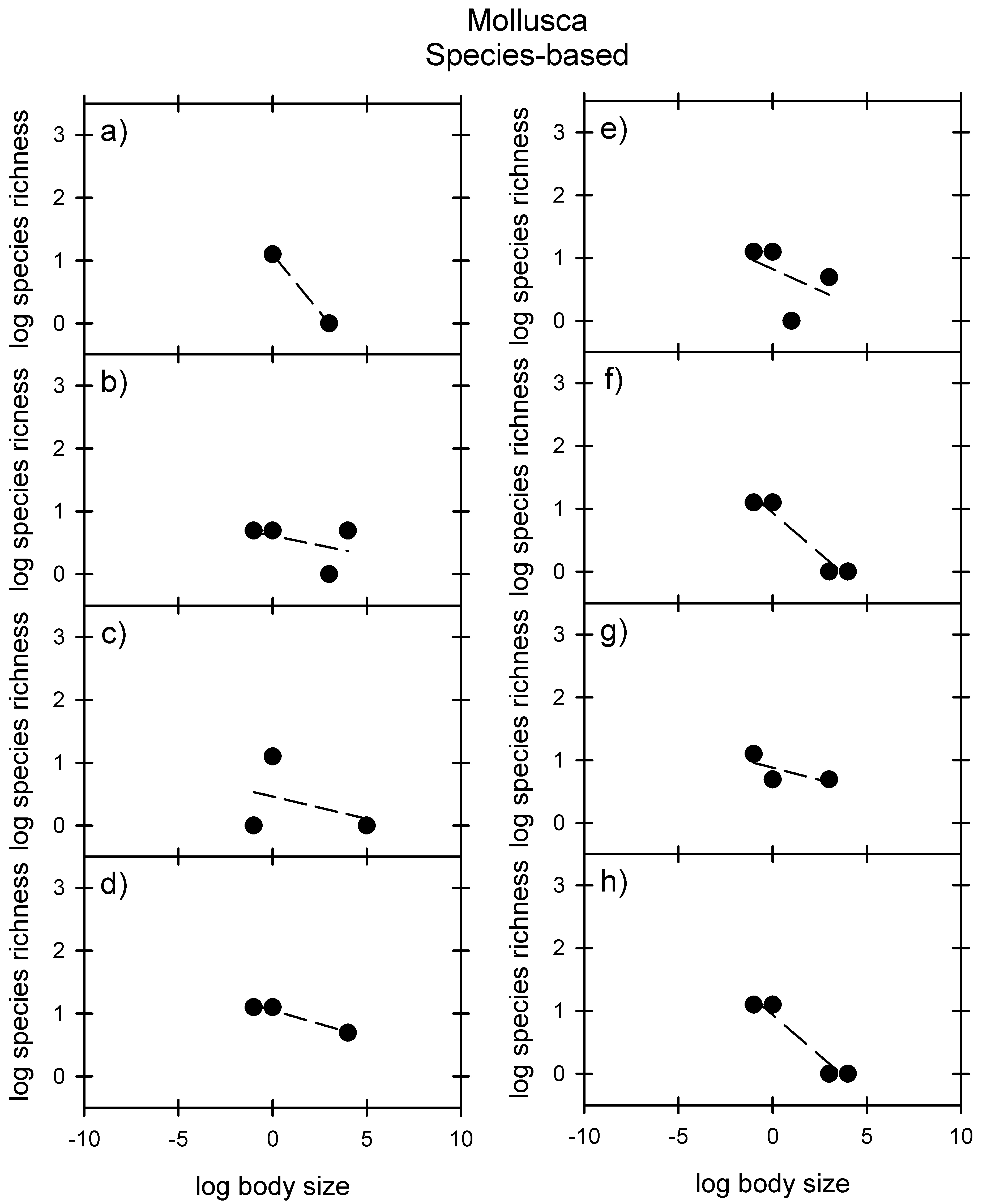 |
| --- |
| **Figure S13:** Temporal dynamics in the relationship between log species richness and species averaged log body size classes in Phylum Mollusca. Figures a to d show the observed values and fitted power law (dashed lines) log-Weibull functions (continuous curves) for log abundance in January, May, August and November 2007 respectively, while figures e to h show the observed values and corresponding fitted functions for January, April, July and October 2008, respectively. Parameter values and fitted R^2^ values are shown in Supporting Table S7, Appendix S1. |

| 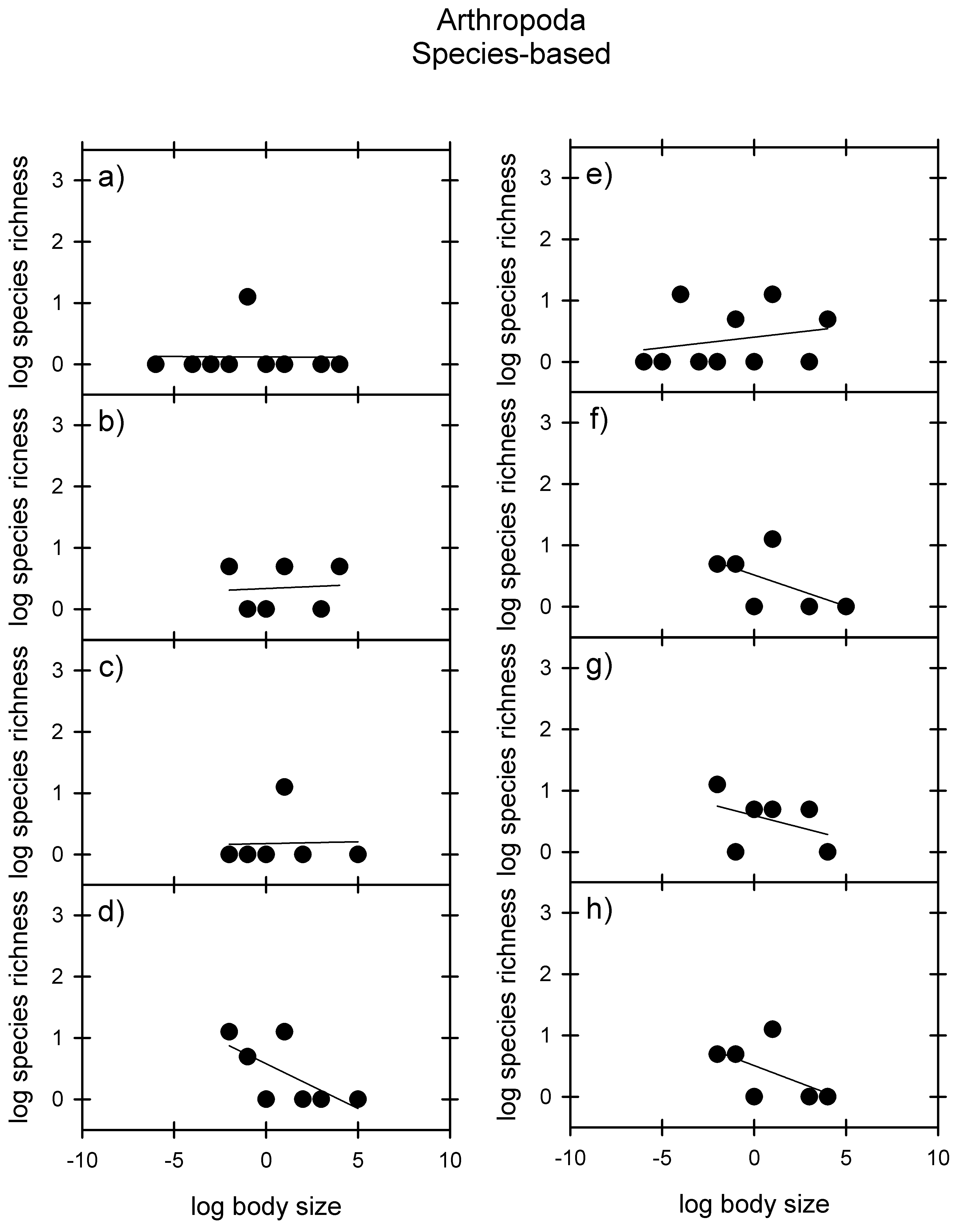 |
| --- |
| **Figure S14:** Temporal dynamics in the relationship between log species richness and species averaged log body size classes in Phylum Arthropoda. Figures a to d show the observed values and fitted power law (dashed lines) log-Weibull functions (continuous curves) for log abundance in January, May, August and November 2007 respectively, while figures e to h show the observed values and corresponding fitted functions for January, April, July and October 2008, respectively. Parameter values and fitted R^2^ values are shown in Supporting Table S7, Appendix S1. |

| 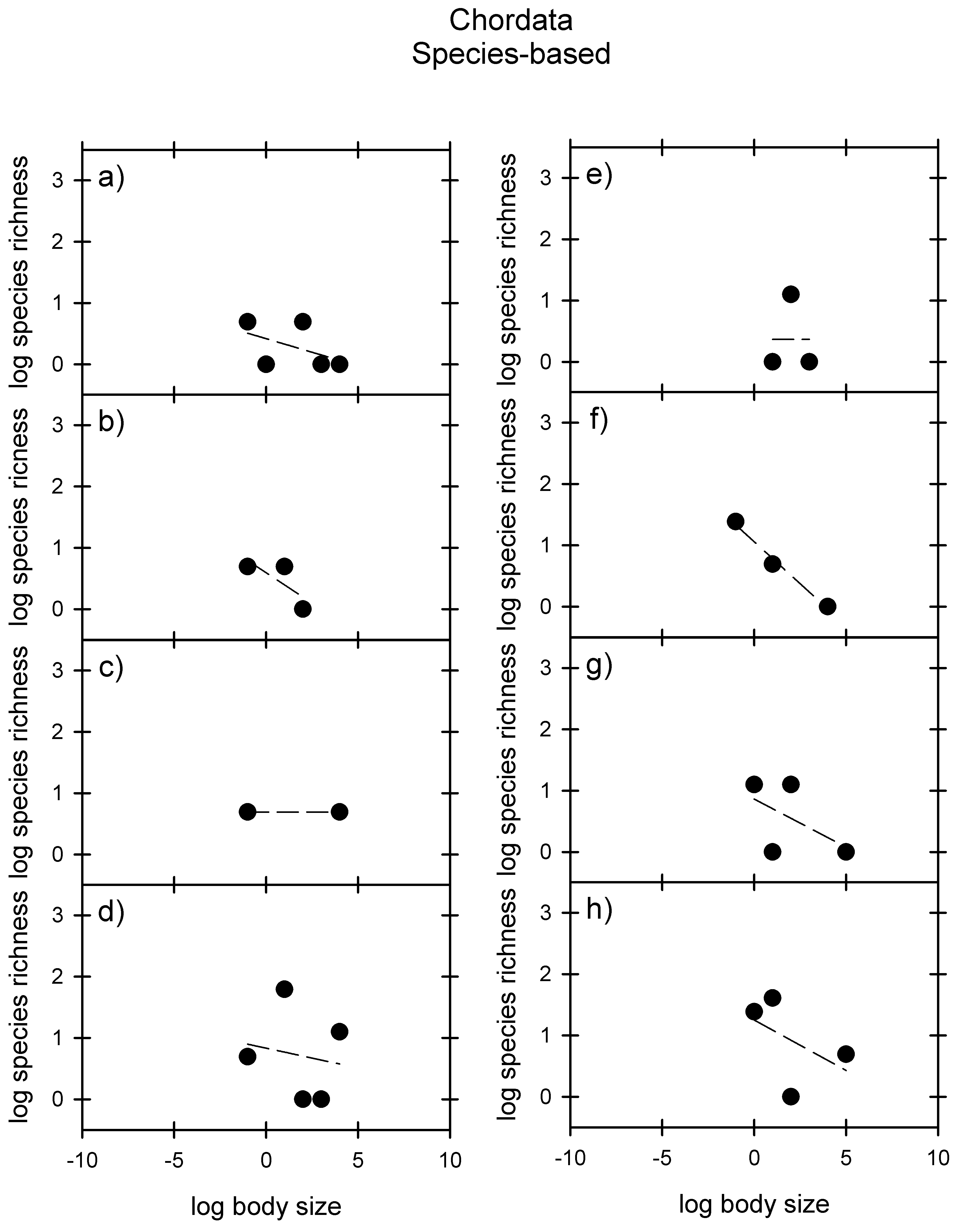 |
| --- |
| **Figure S15:** Temporal dynamics in the relationship between log species richness and species averaged log body size classes in Phylum Chordata. Figures a to d show the observed values and fitted power law (dashed lines) log-Weibull functions (continuous curves) for log abundance in January, May, August and November 2007 respectively, while figures e to h show the observed values and corresponding fitted functions for January, April, July and October 2008 respectively. Parameter values and fitted R^2^ values are shown in Supporting Table S7, Appendix S1. |

| 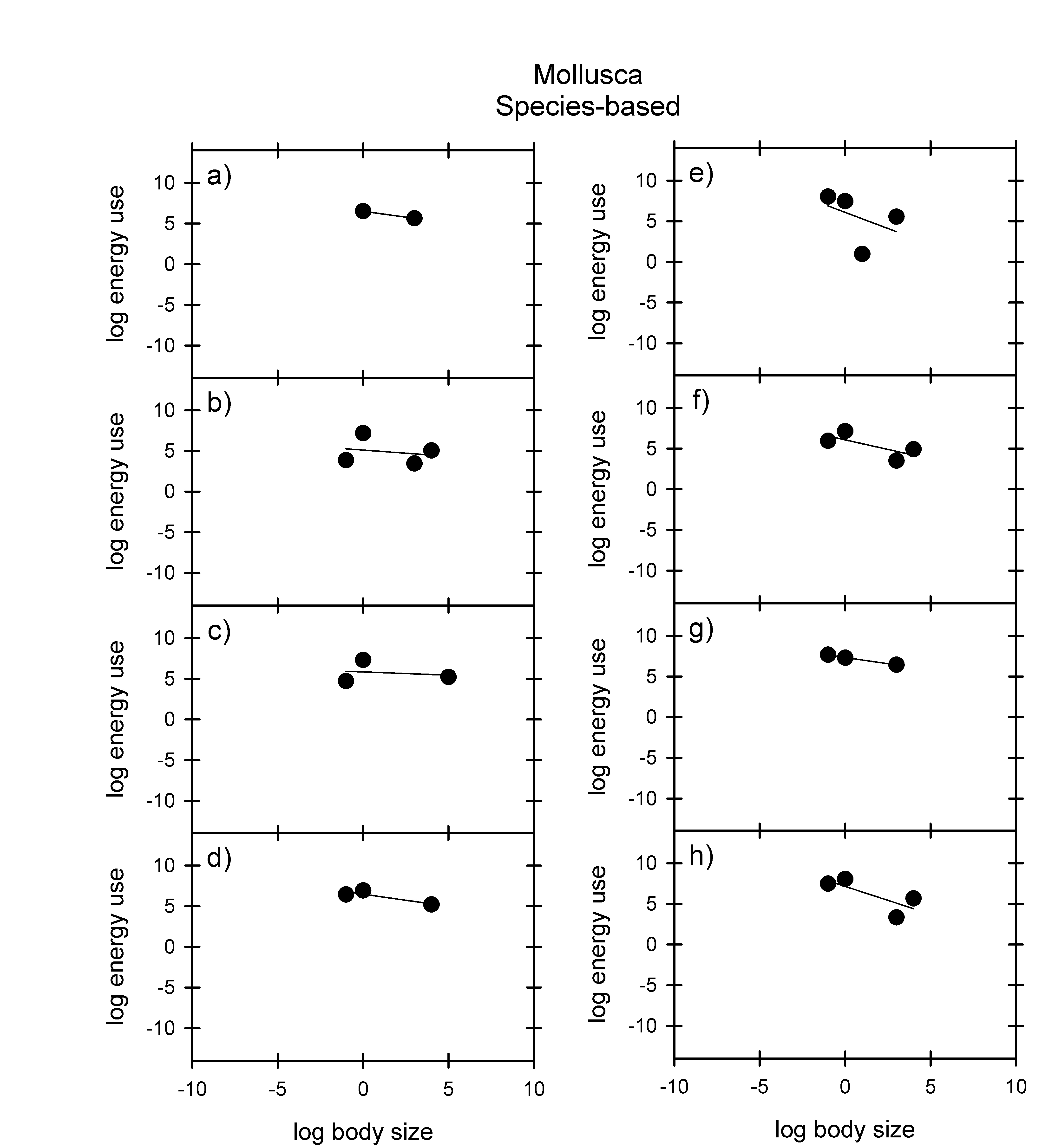 |
| --- |
| **Figure S16:** The figure shows temporal dynamics of the observed scaling of log total energy use (Watts) as a function of species averaged log body size classes in Phylum Mollusca. Filled circles show the total energy use in each body size class. Continuous lines show the best fitted regressions. Figures a to d show the observed values and fitted functions for log energy use in January, May, August and November 2007 respectively, while figures e to h show the observed values and fitted functions for January, April, July and October 2008, respectively. Parameter values and fitted R^2^ values are shown in Supporting Table S8, Appendix S1. |

| 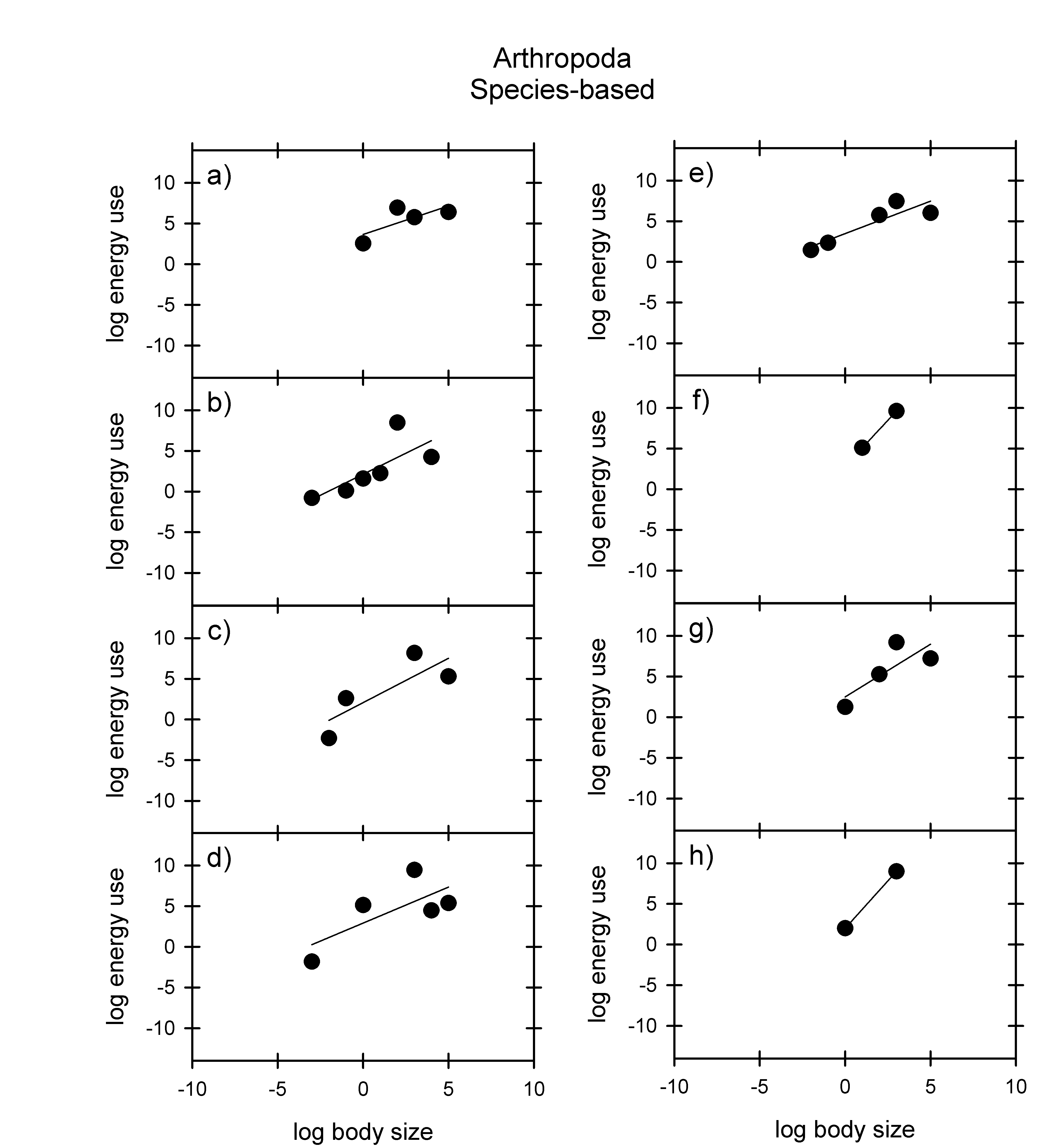 |
| --- |
| **Figure S17:** The figure shows temporal dynamics of the observed scaling of log total energy use (Watts) as a function of species averaged log body size classes in Phylum Arthropoda. Filled circles show the total energy use in each body size class. Continuous lines show the best fitted regressions. Figures a to d show the observed values and fitted functions for log energy use in January, May, August and November 2007 respectively, while figures e to h show the observed values and fitted functions for January, April, July and October 2008, respectively. Parameter values and fitted R^2^ values are shown in Supporting Table S8, Appendix S1. |

| 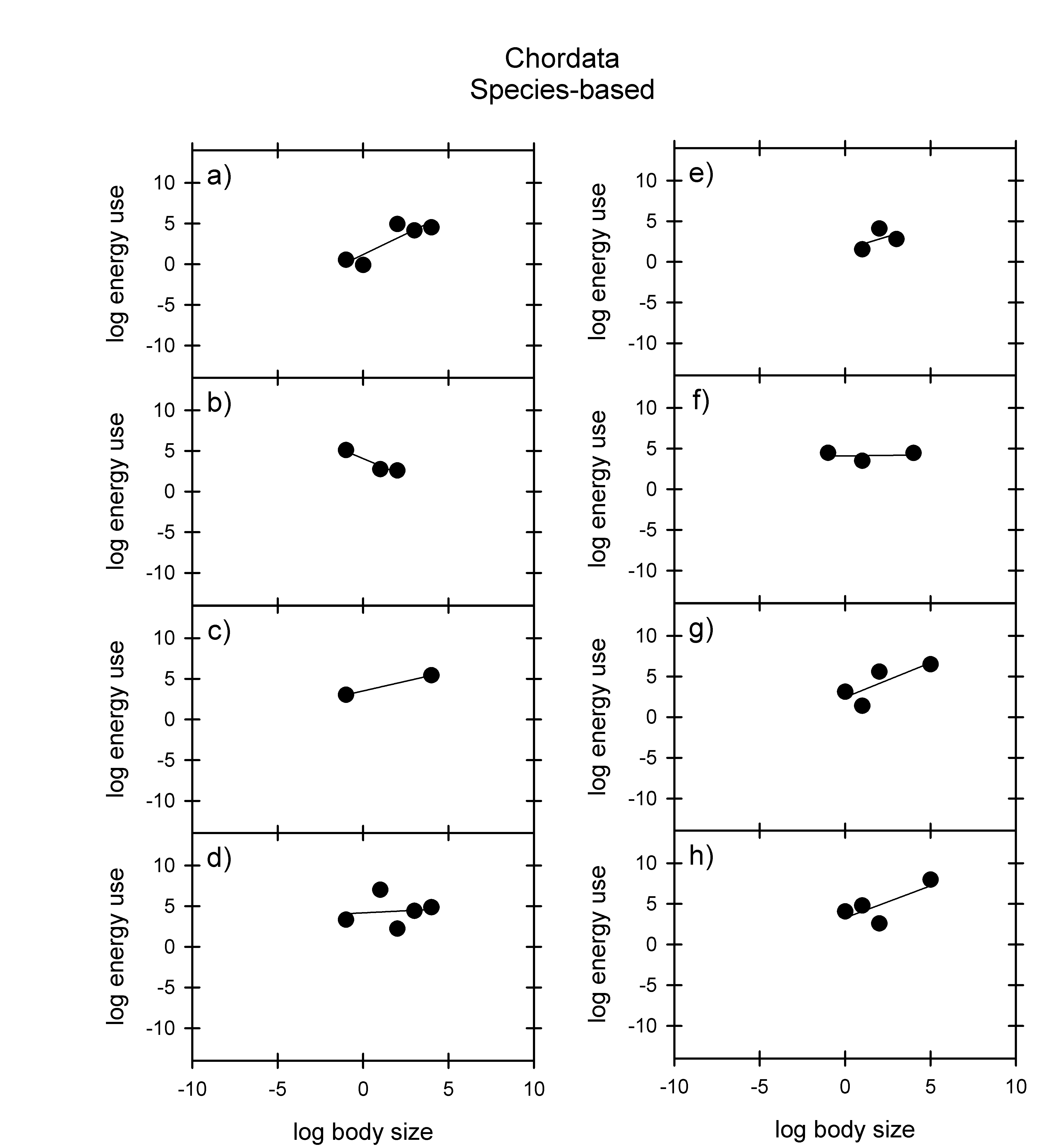 |
| --- |
| **Figure S18:** The figure shows temporal dynamics of the observed scaling of log total energy use (Watts) as a function of species averaged log body size classes in phylum Chordata. Filled circles show the total energy use in each body size class. Continuous lines show the best fitted regressions. Figures a to d show the observed values and fitted functions for log energy use in January, May, August and November 2007 respectively, while figures e to h show the observed values and fitted functions for January, April, July and October 2008, respectively. Parameter values and fitted R^2^ values are shown in Supporting Table S8, Appendix S1. |
